# A comprehensive SPET-based linkage mapping for berry texture and defective seed development in grapevine

**DOI:** 10.3389/fpls.2026.1804241

**Published:** 2026-05-15

**Authors:** Chiara Biselli, Agostino Fricano, Manna Crespan, Simone Giacosa, Simone Garavelloni, Filippo Maria Valentini, Anna Maria Epifani, Francisco Emmanuel Espinosa-Roldán, Luca Rolle

**Affiliations:** 1 Council for Agricultural Research and Economics – Research Centre for Forestry and Wood (CREA-FL), Arezzo, Italy; 2 Council for Agricultural Research and Economics – Research Centre for Genomics and Bioinformatics (CREA-GB), Fiorenzuola d’Arda, Piacenza, Italy; 3 Council for Agricultural Research and Economics – Research Centre for Viticulture and Enology (CREA-VE), Conegliano, Treviso, Italy; 4 Department of Agricultural, Forest, and Food Sciences, University of Torino, Alba, Cuneo, Italy; 5 Council for Agricultural Research and Economics – Research Centre for Viticulture and Enology (CREA-VE), Arezzo, Italy; 6 Council for Agricultural Research and Economics – Research Centre for Forestry and Wood (CREA-FL), Casale Monferrato, Alessandria, Italy; 7 Instituto Madrileño de Investigación y Desarrollo Rural, Agrario y Alimentario (IMIDRA), Alcalá de Henares, Madrid, Spain

**Keywords:** berry texture, BLUPs, in silico gene analysis, phenotyping, QTL mapping, seed morphology, SNP markers, SPET

## Abstract

An F_1_ population of 200 individuals derived from the cross between Raboso Veronese, a black-berried, seeded winegrape cultivar, and Sultanina, a white-berried, seedless table grape cultivar, was phenotyped for berry color and weight, 28 texture-related traits, seed number, and 11 seed size and morphology parameters, and genotyped through Single Primer Enrichment Technology (SPET) to dissect the genetic architecture of berry texture and defective seed development. High-density pseudo-testcross genetic maps were obtained for both parents, comprising, respectively, 1, 223 and 1, 238 SNP markers. Quantitative trait locus (QTL) analysis, performed using BLUPs of phenotypic data, revealed 12 closely located QTLs in Sultanina chromosome 18 associated to berry texture and/or seed traits, with LOD values up to 45 and explained phenotypic variation up to 65.49%. In addition, several minor-effect QTLs related to berry traits were detected: on chromosomes 11, 17, and 18 in Raboso Veronese, and on chromosome 11 in Sultanina. Although each locus individually explained a limited proportion of phenotypic variance, their combined effects supported the polygenic nature of berry mechanical properties. *In silico* analysis of genes within the identified QTL regions revealed functions consistent with roles in the genetic regulation of berry texture and seed development status, supporting the association of these regions with the observed phenotypic variation and providing a foundation for future in depth functional studies. Thanks to the accurate joint phenotyping of berry texture and seed development deficiency, the two parental maps allowed us to highlight several aspects of grape texture dynamics, underlining the link between seed development status and berry softening during ripening.

## 1 Introduction

Berry mechanical properties (or berry texture) and seedlessness are complex, pivotal traits influencing grapevine fruit quality, consumer preference, and suitability for various end-uses such as table consumption, winemaking, and raisin production. From this point of view, wine grapes and table grapes differ notably: the first are smaller, have softer flesh and thicker skin; they have also large seeds containing tannins which play an important role in wine organoleptic characteristics and ageing potential. On the other hand, table grapes characterized by seedless and crisp berries are preferred by consumers (Kuhn et al., 2014).

Mechanical properties are related to grape variety, berry ripening level, growing location, and climatic conditions of the season (Rolle et al., 2012). Particularly for red winegrape varieties, berry skin break force (F_sk_) is related with yield and extraction of anthocyanins and tannins during maceration, in which hard skins are characterized by slower release of polyphenols and lower yield (Rolle et al., 2008). In parallel, in grape varieties characterized by high F_sk_ values, the kinetics of dehydration during the withering process showed a lower daily weight loss under controlled operating conditions of temperature, relative humidity and air flow (Giacosa et al., 2012). Skin thickness (Sp_sk_) is an important parameter for both winegrapes and table grapes: in the first case there is an inverse correlation with anthocyanin release during maceration (Río Segade et al., 2011), while on table grapes a higher value induces a lower sensory acceptance by consumers but higher shelf life and damage resistances (Rolle et al., 2013). Whole firmness and pulp berry texture characteristics (Texture Profile Analysis variables - TPA) are mechanical properties highly important for table grapes because they are directly related to berry crunchiness, affecting the quality perceived by consumers (Giacosa et al., 2015).

Several QTL mapping and genome-wide association studies (GWAS) have identified regions influencing berry texture, seed weight, seed number, and related traits in both, table and winegrape cultivars (Doligez et al., 2002, Doligez et al., 2013; Carreño et al., 2015; Lin et al., 2023; Hu et al., 2025). These studies have highlighted the polygenic nature of these traits and their partial QTL overlap, but there are still gaps in knowledge. Based on the most reliable theory (Bouquet and Danglot, 1996), the defective seed development in grapevine should depend by a dominant gene, nowadays recognized in *VviAGL11* (Mejía et al., 2007; Costantini et al., 2008), plus three independent and recessive genes, controlled by the major gene, which have not yet been identified. On the other hand, the numerous studies conducted so far on berry texture provided a still fragmented frame. We decided to address both issues together, given the availability of an F_1_ population segregating for both berry texture and defective seed development traits.

Among candidate genes, the MADS-box transcription factor encoding gene *VviAGL11* has been functionally validated as a major regulator of fruit set and expansion, and seedlessness (Royo et al., 2018; Song and Chen, 2018; Pi et al., 2021; Wang et al., 2024). Moreover, it represented the best candidate locus underlying a major QTL for berry texture in an F_1_ population derived by crossing the seedless Sultanina with the wine cultivar Raboso Veronese (Crespan et al., 2021). In this population, this QTL explained more than 60% of phenotypic variation for berry firmness, suggesting a link between the two traits.

Despite the relevance of berry texture and seedlessness for both wine and table grape production, the genetic bases underpinning these traits remain incompletely understood, primarily due to their complex inheritance patterns and the challenges associated with precise phenotyping. For this reason, a better understanding of the genetic control of these traits could contribute to better selecting desirable characteristics in plant breeding programs and may even help the agronomic management of grapevines according to production purposes. Alongside the complex genetic regulation of such traits, their dissection is further complicated by the challenges of obtaining precise, high-resolution phenotypic measurements.

Linkage mapping has been instrumental in investigating complex traits and the current high-throughput genotyping technologies enable the construction of dense genetic maps for precise QTL mapping. Single Nucleotide Polymorphism (SNP) arrays and Genotyping−by−Sequencing (GBS) have been widely applied to grapevine, producing thousands of markers for linkage mapping (Teh et al., 2017; Tello et al., 2019; Vervalle et al., 2022). However, the marker density and distribution generated with these methodologies are often limited and can affect map quality and QTL resolution, particularly in highly heterozygous species like grapevine (Rasheed et al., 2017; Frenzke et al., 2024).

Single Primer Enrichment Technology (SPET) has emerged as a powerful method for targeted genotyping, with costs comparable to SNP arrays but greater flexibility and can potentially overcome the limitations of other genotyping methodologies. SPET combines the choice of targeted marker selection with the advantages of amplicon-based sequencing at high multiplexing capabilities that improve marker density and distribution uniformity, in comparison to GBS or SNP arrays. It allows flexible and efficient genotyping that leads to *de novo* polymorphism discovery (Barchi et al., 2019; Scaglione et al., 2019). In *Vitis vinifera*, SPET-based genotyping has allowed to generate dense polymorphism datasets, including not only SNPs but also Insertion/Deletions (In/Dels), and to capture genetic variation across cultivars and wild relatives (Marinov et al., 2024). Even with only limited examples, SPET has been utilized also for linkage mapping approaches in oil palm (Herrero et al., 2020) and *Plasmopara viticola* (Dvorak et al., 2025).

In the present study we combined SPET-based high-throughput linkage mapping with accurate phenotyping for berry texture and seed development status, using the same F_1_ population (Raboso Veronese × Sultanina) utilized by Crespan et al. (2021), including 200 individuals segregating for these traits The previous work on this population was limited by SSR-based mapping, with 158 mapped markers, and by searching only for texture-associated QTLs on the consensus map (Crespan et al., 2021). Going beyond those previous results, we performed a comprehensive automated precise phenotyping including the evaluation of berry weight, 28 traits related to berry texture, seed number, and 11 parameters related to seed weight and size. Notably, the size of seeds in the population showed a continuous variability, ranging from normally developed to progressively herbaceous and stunted, up to the complete absence. Despite some limitations inherent to SPET genotyping, the integration of high-density genotypic and detailed phenotypic data revealed multiple QTLs controlling berry and seed traits. Known and novel loci were found, carrying genes with annotated functions and supporting the roles of the detected genomic regions in the genetic control of these traits. To our knowledge, this is the first time that such detailed analyses have been combined in the same study, providing new insights into the genetic mechanisms underlying complex traits, that may be exploited in breeding programs aimed at improving grapevine fruit quality.

## 2 Materials and methods

### 2.1 Plant material and phenotyping

An F_1_ population of 200 individuals, already described in a previous linkage mapping study (Crespan et al., 2021) and obtained by crossing the black-berried, seeded winegrape Raboso Veronese and the white-berried, seedless table grape Sultanina, was used for this study. Five vines per seedling were grafted on Kober 5BB and planted in 2012 at CREA Viticulture and Enology experimental farm in Spresiano (Treviso, Italy, geographic coordinates 45° 47′ N, 12° 15′ E).

#### 2.1.1 Berry traits

Thirty berry traits were considered in this study and are reported in **Table 1**.

**Table 1:** List of the berry traits evaluated in this study with the corresponding abbreviations.

Berry trait	Abbreviation
Berry Color	BC
Average Berry Weight	ABW
Berry Resilience	BR
Berry Resilience normalized on berry diameter	BR_diam
Berry Resilience normalized on berry surface	BR_sur
Berry Resilience normalized on berry volume	BR_vol
Berry Springiness	BS_ratio
Berry Springiness normalized on berry diameter	BS_ratio_diam
Berry Springiness normalized on berry surface	BS_ratio_sur
Berry Springiness normalized on berry volume	BS_ratio_vol
Berry Cohesiveness	BCo
Berry Cohesiveness normalized on berry diameter	BCo_diam
Berry Cohesiveness normalized on berry surface	BCo_sur
Berry Cohesiveness normalized on berry volume	BCo_vol
Berry Hardness	BH
Berry Hardness normalized on berry diameter	BH_diam
Berry Hardness normalized on berry surface	BH_sur
Berry Hardness normalized on berry volume	BH_vol
Berry Gumminess	BG
Berry Gumminess normalized on berry diameter	BG_diam
Berry Gumminess normalized on berry surface	BG_sur
Berry Gumminess normalized on berry volume	BG_vol
Berry Chewiness	BCh
Berry Chewiness normalized on berry diameter	BCh_diam
Berry Chewiness normalized on berry surface	BCh_sur
Berry Chewiness normalized on berry volume	BCh_vol
Berry skin break force	F_sk_
Berry skin break energy	W_sk_
Berry skin resistance to the axial deformation	E_sk_
Berry skin thickness	Sp_sk_

For each genotype (F_1_ progeny and parents), berry color has been visually determined based on presence/absence of anthocyanins. Average berry weight was measured on 50 unfloated berries, differentially from previous analyses conducted in 2017 and 2019 in which 20 unfloated berries were used (Crespan et al., 2021).

In 2022, 28 traits related to berry mechanical properties (**Table 1**) have been evaluated, applying Texture Analysis tests (cluster sampling, puncture test, compression test, texture profile analysis test, TPA) with operative conditions (probe, speed test, load cell, data acquisition) described in detail previously in Crespan et al. (2021). The data has been integrated with the ones recorded in 2017 and 2019 (Crespan et al., 2021). In brief, berries with attached pedicels from each genotype were subjected to density sorting by flotation in saline solutions presenting concentrations of 110, 120, 130, 140, 150 g/L NaCl, selecting berries belonging to a density class of 1088 kg/m^3^ (130 g/L NaCl solution). The texture parameters were determined for each genotype us) andTA.XTplus universal testing machine (Stable Micro Systems, Godalming, Surrey, United Kingdom) considering berry skin breakage (Letaief et al., 2008), berry skin thickness (Río Segade et al., 2011) and TPA on whole berry (Rolle et al., 2012), with berry springiness and chewiness parameters modified by Crespan et al. (2021).

#### 2.1.2 Seed traits

Twelve seed traits were measured, as reported in **Table 2**.

**Table 2:** List of the seed traits evaluated in the present study with the corresponding abbreviations.

Seed trait	Abbreviation
Seed number	NS
Fresh Seed Weight	FSW
Dried Seed Weight	DSW
Ratio between FSW and DSW	FSW/DSW
Feret	F
Minimum Feret	MF
Ratio between Feret and Minimum Feret	Feret AR
Perimeter	P
Area	A
Circularity	Circ
Roundness	R
Solidity	S

In 2019 and 2020, for the F_1_ individuals and Raboso Veronese, from three to five bunches were randomly harvested at maturity and processed at CREA-VE laboratory in Susegana (TV, Italy). Twenty-five berries located in the middle of the bunch were weighed and dissected to evaluate the presence of seeds, when possible. Recovered seeds included both normally developed and lignified ones and those herbaceous or partially lignified, small, shriveled or sketched. Seed number (NS) refers to the total number of normally developed lignified seeds in the 25 berries. All seeds were cleaned and washed, dried with paper, and weighed to obtain fresh seed weight (FSW). Seeds were then dried in oven at 80 °C for three days and weighed again to get dried seed weight (DSW).

**Figure 1:**
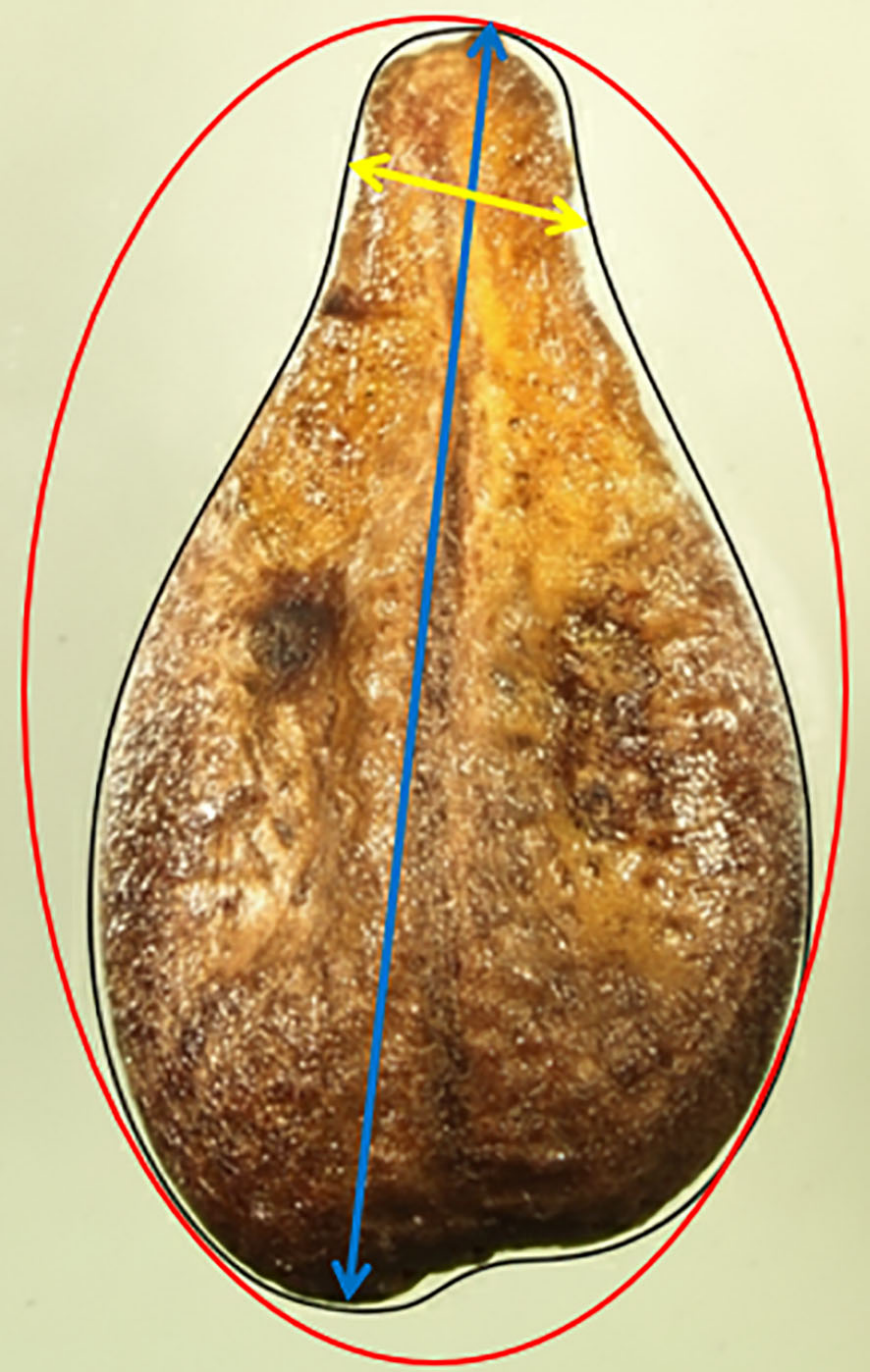
Schematic representation of the base parameters that characterize seed shape obtained using the ImageJ software. Blue and yellow arrows indicate respectively feret (F) and minimum feret (MF), perimeter (P) is shaped in black, and the red line encloses the convex area used to calculate solidity (S).

The traits related to seed dimension and shape were measured as indicated by Cervantes et al. (2021), at CREA-VE laboratory in Arezzo (Italy). For each genotype, all the collected seeds from the 25 berries were positioned on microscopy glass slides, with the chalaza downwards to expose the ribs upwards, straight in the middle and vertically oriented. Each microscopy glass slide was photographed by a Canon EOS 700D camera (Canon Inc., Ōta, Tokyo, Japan), and, for each seed, seed parameters were extrapolated by analyzing the pictures using the ImageJ 1.54d software (Ferreira and Rasband, 2011, accessed on July 3, 2025). In detail, Feret (F) describes the maximum distance between two points on the outline of an object; Minimum Feret (MF) is the minimum distance between two points on the outline of an object (**Figure 1**). Perimeter (P) was calculated as the length of the object boundary; Area (A) was determined as the sum of all pixels enclosed by the object boundary. Circularity, Roundness and Solidity were calculated according to the following equations:

Circularity, a shape descriptor that quantifies how close the seed outline is to a perfect circle.

(1) $$Circ=4\Pi A/P^{2}$$

Roundness quantifies how close the seed outline is to a circular shape.

(2) $$R=4A/\Pi F^{2}$$

Solidity measures the compactness of the seed outline, indicating the proportion of the convex area occupied by the seed.

(3) S=A/convex A

In Equation 3, convex area (convex A) refers to the area of the convex hull of an object, that represents the minimum enclosing polygon that completely encloses its outline (**Figure 1**).

#### 2.1.3 Statistical analysis of phenotypic data

Raw phenotypic data of berry and seed traits showing values higher or lower than 5 standard deviations from the corresponding means were identified as outliers and removed from subsequent analyses. The resulting raw phenotypic data of seed and texture traits were examined to compute the corresponding adjusted means using the following linear mixed model:

(4) $$y_{jk}=1µ+Gen_{j}+Year_{k}+e_{jk}$$

where *y_ik_* is the response variable, that is one of the texture or seed traits reported in **Table 1** or **Table 2**, µ is the general mean, *Year_k_* is the fixed effect of the *k^th^* year in which the observations were collected, *Gen_j_* is the random effect of the *j^th^* genotype, which was assumed to follow a multivariate normal distribution with mean 0 and unstructured covariance matrix, that is $Gen_{j}\sim N\left(0,\text{Σ}\right)$, and *e_jk_* is the error associated to each response, which was supposed to be independent and identically distributed, that is $e_{jk}\sim N\left(0,\sigma_{e}^{2}\right)$. Interaction of genotype x year was firstly considered as random effect, but it was later dropped from the final model (Equation 4) using backward selection of predictors as this term showed unsignificant P-value (>0.05) in the full model for all traits examined in the present study. Model assumptions were verified examining the distribution of random effects and residuals obtained after fitting the models.

The abovementioned linear mixed models used for computing the Best Linear Unbiased Predictors (BLUPs) of genotypic values were fitted using lme4 package (Bates et al., 2015) in R 4.1.3 statistical environment (R Core Team, 2022). BLUPs of genotypic effects computed for all traits examined in the present study were combined with genetic map information to carry out QTL analyses.

Pearson’s correlation coefficients showing *P-*values< 0.05 were computed between adjusted means (BLUPs) of grape and seed traits showing continuous variations, while polyserial correlations were computed to measure correlations between continuous traits and BC which was scored as an ordinal trait assuming values 1 (white berry) or 2 (black berry) (Drasgow, 2014). All correlation analyses were computed using “polycor” package (Fox, 2022, accessed on January 19, 2026) implemented in R 4.1.3 statistical environment (R Core Team, 2022). A Principal Component Analysis was performed on berry data using R and the “factoextra” package (R Core Team, 2022; Kassambara and Mundt, 2020; both accessed on February 4, 2026).

### 2.2 Genotyping

DNA extraction and SPET genotyping were performed at IGATech (IGA Technology Services s.r.l., Parco Scientifico e Tecnologico “L. Danieli”, via J. Linussio 51 Z.I.U., 33100 Udine, Italy). For each genotype, including the parents Raboso Veronese and Sultanina, genomic DNA was purified from 100 mg of grinded young leaves using the Mag-Bind^®^Plant DNA DS Kit (Omega Bio-Tek Inc, Norcross, Georgia, USA), according to manufacturer’s instructions with minor modifications (2% PVP and 1% beta-mercaptoethanol added in the lysis buffer). DNAs were quantified using Qubit 2.0 Fluorometer (Invitrogen, Carlsbad, CA).

Genotyping was based on the SPET system developed by IGATech and including 50, 000 grapevine genic regions that generated a half million SNPs from Croatian grapevine germplasm (Marinov et al., 2024). To maximize resolution and variant calling at the SPET loci, the two parents were subjected to Whole Genome Shotgun sequencing. Libraries were prepared by the ‘Ovation Ultralow System V2 DNA-Seq’ kit (NuGEN, San Carlos, CA, USA), following manufacturer’s instructions, quantified by Qubit 2.0 Fluorometer (Invitrogen, Carlsbad, CA, USA), and quality tested by Agilent 2100 Bioanalyzer High Sensitivity DNA assay (Agilent technologies, Santa Clara, CA, USA). The sequencing was conducted on Illumina NovaSeq 6000 (Illumina, San Diego, USA) in paired-end 150 bp mode and the data were subjected to the SPET bioinformatic pipeline.

For the F_1_ progeny, SPET library preparation, sequencing, and bioinformatic pipeline (base calling, quality trimming, alignment to the reference, and discovery of allelic variants) were conducted according to the IGATech standard protocols for SPET described in Marinov et al. (2024). *V. vinifera* 12x.v0 genome (http://vitisgdb.ynau.edu.cn/downloads.html) was used as reference genome. Briefly, a locus was considered when at least 150 of the samples displayed more than 10X coverage.

### 2.3 Map construction and QTL analysis

Linkage mapping was performed using the pseudo-testcross method (Grattapaglia and Sederoff, 1994), running the R package OneMap for outcrossing species (Margarido et al., 2007). The two linkage maps, one for each parent, were independently built up following the steps for high resolution mapping reported in literature (Herrero et al., 2020; Possamai et al., 2021). For map construction, molecular markers showing segregation ratios of 1:1:1:1 (full cross segregation), 1:1 (test cross segregation), and 1:2:1 (F_2_ ratio, with one allele in common in the two parents) were selected and were separated on the basis of the parent from which each marker segregates. By this way, two different datasets, one for each parental genotype, were created.

For each parental dataset, markers were filtered for Minimum Allele Frequency (MAF) > 10%, redundancy (no recombination events between each other; binning using default parameters), and segregation distortion (SD). SD was assessed using the χ^2^ test with *P*-values< 0.005. Calculations were performed using the OneMap default parameters for outcrossing populations, which apply a global α = 0.05 threshold and include a Bonferroni correction for multiple testing. The recombination fraction (rf) between all pairs of markers was calculated using two-point tests. Linkage groups (LGs) were identified using a LOD threshold equals to 8.0 and a rf threshold of 0.30. Marker order was obtained by means of Kosambi mapping function, adjusted with the OneMap implementation including information on reference genome. Each LG order was manually checked to eliminate markers in weak linkage (low mean association-LOD value/high rf) with the neighboring markers and to adjust marker order according to the most significant rf. Kosambi algorithm was also used to calculate marker distances. The resulting genetic maps were projected to grapevine reference genome 12x.v0 (http://vitisgdb.ynau.edu.cn/downloads.html) to find possible rearrangements with respect to the reference sequence. For each LG in the two maps, Spearman correlation analyses between genetic and annotated physical positions at each marker were conducted to evaluate the collinearity with the reference genome.

QTL analysis was performed separately on Raboso Veronese and Sultanina genetic maps by the R/qtl package (Broman et al., 2003; Broman and Sen, 2009). Thirty-three parameters out of 42 were analyzed independently, excluding normalized BR, BS_ratio, and BCo, because resulted not affected by berry size after phenotypic data correlation clustering. Firstly, a single-trait QTL analysis was applied using the Haley-Knott regression method and genome-wide LOD significance threshold calculated by permutation tests (1000 permutations and *P*-values< 0.01). This first step of analysis was then complemented by a multi-trait approach in which the 33 phenotypic traits were mined simultaneously to detect loci with shared genetic effects and to confirm the results of individual trait scans. Even in this case 1000 permutations and *P*-values< 0.01 were applied.

For each QTL, Bayes confidence intervals (CIs) were determined at a significance probability level of 0.95. Epistatic effects between QTLs associated with the same trait in the same genetic background were mined by the Multiple QTL Mapping method. The Haley-Knott regression method and genome-wide LOD significance threshold calculated by permutation tests (1000 permutations and *P*-values< 0.01) were utilized. Additive effects of detected QTLs were extracted with R/qtl package.

### 2.4 *In silico* functional analysis of annotated genes inside the confidence intervals of the detected QTLs

*In silico* functional analysis of annotated genes located within the CIs of the detected QTLs was conducted to find out functions related to the regulation of the phenotypic traits considered in this study. To identify functions potentially related to the regulation of berry texture, genes involved in cell wall organization and remodeling, sugar transport, and hormone signaling and regulation were considered. For seed size and development, genes implicated in ovule and seed coat formation, embryogenesis, and hormone-mediated growth were targeted.

While SPET genotyping and linkage mapping were performed using the grapevine genome 12x.v0 release, the molecular markers delimiting each CI were projected onto the most recent version of the *V. vinifera* reference genome, PN40024.v5 (https://www.grapegenomics.com/pages/PN40024/), to determine their corresponding physical positions and integrate the QTL data with up-to-date gene annotations. This projection ensures consistency between the QTL intervals and annotated gene coordinates, despite the use of different genome versions. Subsequently, the list of annotated genes within each CI was retrieved from the *V. vinifera* section of the Ensembl Plants database (https://plants.ensembl.org/Vitis_vinifera/Info/Index). Functional information for each gene was then obtained from the InterPro database (https://www.ebi.ac.uk/interpro/).

ChatGPT (OpenAI; ChatGPT standard version, based on the GPT-4 model; https://chatgpt.com) was used, during September and October 2025, only to support literature exploration in Google Scholar for the identification of possible candidate genes within QTL CIs. The model was instructed to retrieve information exclusively from peer-reviewed publications. All outputs were critically evaluated by the authors and integrated with independent manual bibliographic searches. ChatGPT was not involved in any experimental design, data analysis, or gene prediction.

## 3 Results

### 3.1 Phenotyping for berry and seed traits

The 200 individuals of the F_1_ population and the two parents were phenotyped, across multiple seasons, for 42 traits tied to berry color, average berry weight, berry texture, seed number, seed weight, and seed morphology (**Tables 1**, **2**). Adjusted means of raw phenotypic values were calculated and examined to assess the distribution of each trait and to pinpoint groups of correlated parameters.

**Figure 2:**
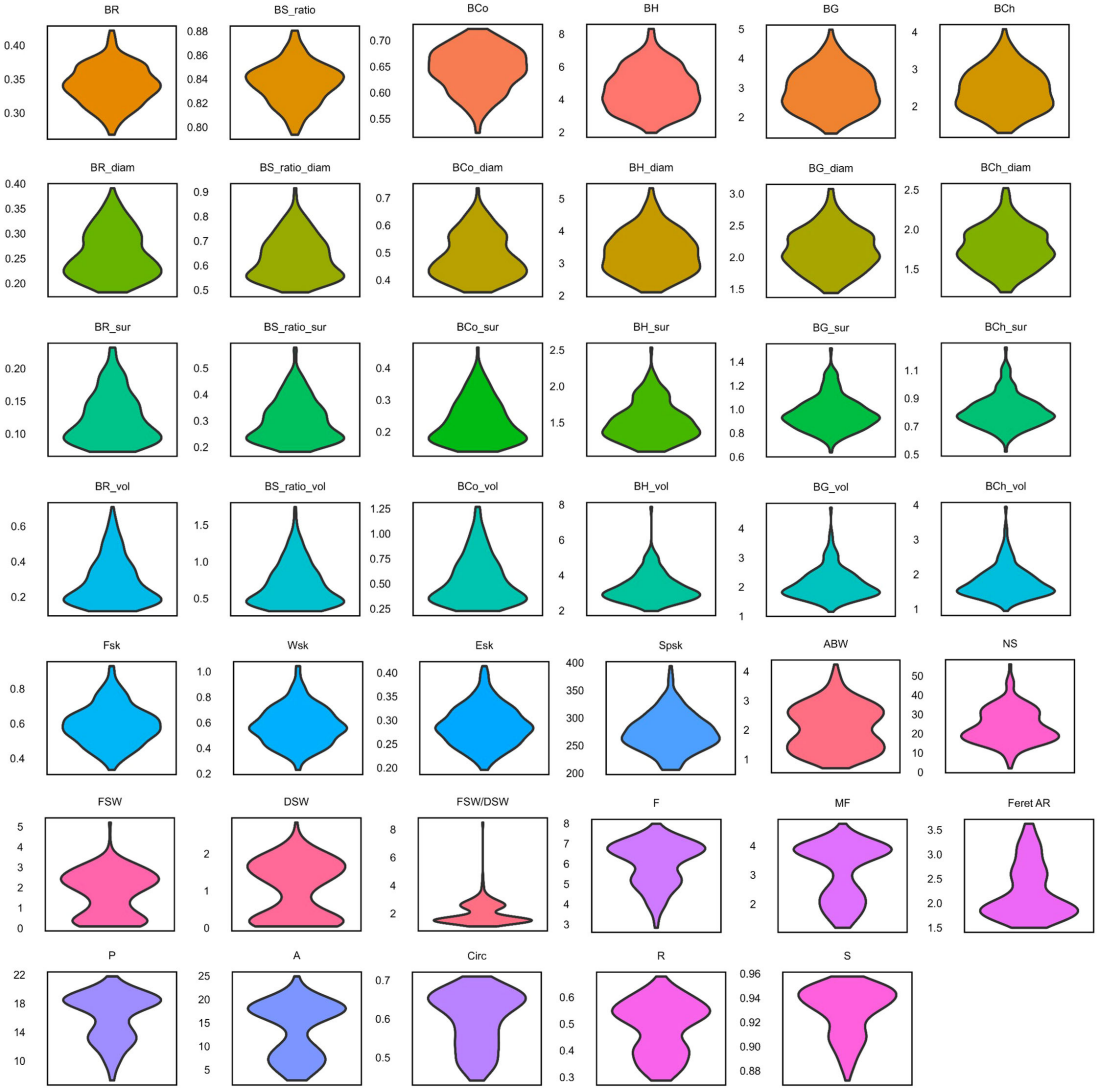
Violin plots show the distribution of adjusted means of seed and berry traits. Each violin plot reports the density plot of the adjusted means computed for each trait. Trait abbreviations reported in each subplot are described in **Tables 1**, **2**.

Density plots of adjusted means showed that almost all berry texture traits have an approximate normal distribution or a skewed distribution. Differently, the density plots of average berry weight (ABW) and the seed traits area (A), perimeter (P), minimum Feret (MF), roundness (R), fresh seed weight (FSW), dried seed weight (DSW), and FSW/DSW showed a bimodal distribution (**Figure 2**).

#### 3.1.1 Correlation clusters

**Figure 3:**
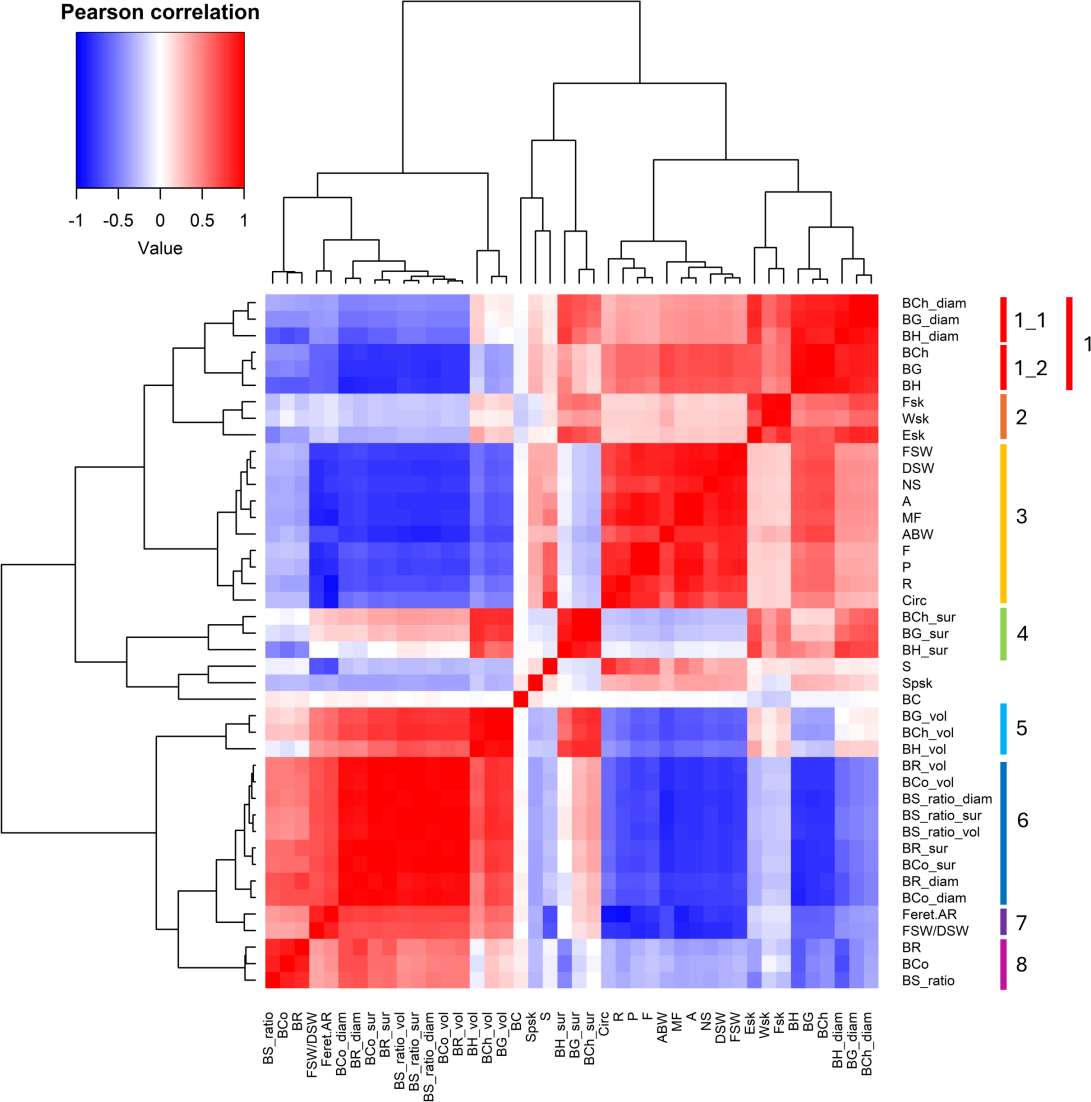
Heatmap of the pairwise correlations between the phenotypic traits measured in the present study. Pairwise Pearson’s correlation coefficients are represented with colour gradients varying from blue (negative correlations) to red (positive correlations), while correlation between berry colour (BC) and other traits were computed using polyserial correlations. Similarly, correlations of the number of seeds (NS) and other traits were computed using polycoric correlation. Correlation Clusters (CCs) are indicated by coloured bars on the right. Pearson correlation scale shown in the upper left; red = positive, blue = negative correlations. Trait abbreviations are described in **Tables 1**, **2**.

Berry and seed-related traits were classified into eight clear Correlation Clusters (CCs) that correspond to distinct groups of traits (**Figure 3**).

Six CCs (1, 2, 4, 5, 6 and 8) were related to berry parameters, while two CCs (3 and 7) were related to seed traits. BC, S, and Sp_sk_ were not included to any CC.

A trend was observed for texture parameters: BH, BG, and BCh grouped in CC1, CC4 and CC5, while BR, BCo, and BS_ratio grouped in CC6 and CC8. Skin mechanical properties grouped in CC2.

CC3 contained most seed parameters (FSW, DSW, NS, A, MF, F, P, R, Circ), and average berry weight (ABW), while CC7 included two ratios: FSW/DSW and Feret AR.

Notably, BR, BCo, and BS_ratio (CCs 6 and 8) showed significant negative correlations with BCh, BG, and BH (CCs 1, 4, and 5), as well as with ABW and the seed traits grouped in CC3. Conversely, CCs 6 and 8 were positively correlated with the seed-related CC7 (FeretAR and FSW/DSW). CC7, in turn, displayed a significant negative correlation with CC3, which was positively associated with BH, BG, and BCh (SubCC1_2), and negatively with BR, BCo, and BS_ratio (CCs 6 and 8), as well as with BG_vol, BH_vol, and BCh_vol (CC5).

The significant levels of pair-wise correlations (positive or negative) recorded between most of the traits considered in the present study agree with previous studies showing that berry shape and seed shape are interdependent, especially in white cultivars (Barbagallo et al., 2020), and that berry weight is more closely related to seed mass than to seed number (Gil et al., 2015). Accordingly, for our genetic resources, heavier berries tend to have heavier and bigger seeds, with a lower relation with seed number, influencing the seed-to-pulp ratio. Moreover, heavier berries showed higher firmness, with higher levels of chewiness, gumminess, hardness and lower resilience, cohesiveness, and springiness. Overall, our correlation analyses, carried out after accounting for potential confounding factors, clearly indicate that some of the traits examined in the present study might be genetically correlated.

#### 3.1.2 PCA

**Figure 4:**
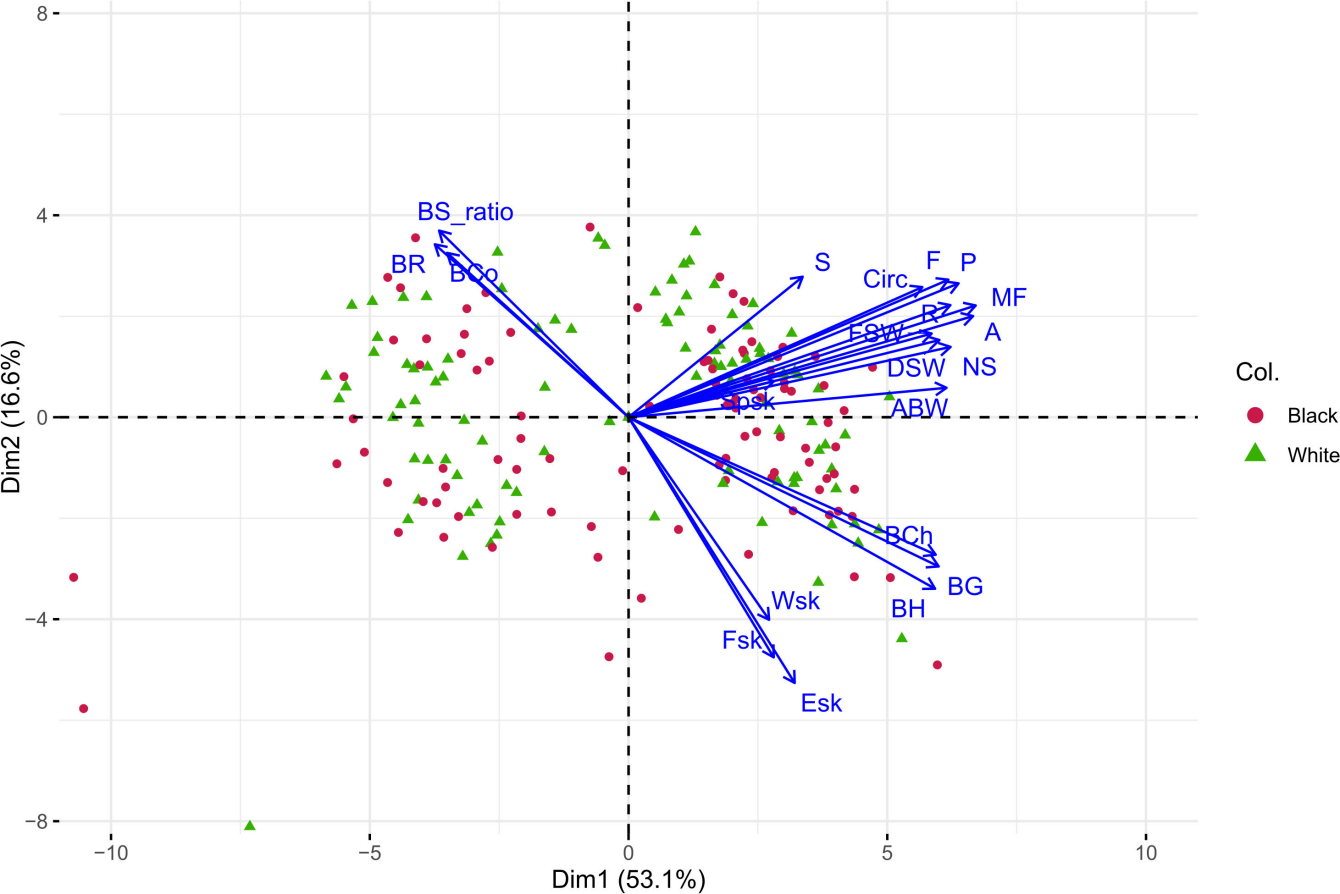
Principal Component Analysis of texture and seed data. Seed and berry texture parameters (whole berry TPA parameters without normalization; berry skin break force and thickness) were considered. Berry color (Col.) is represented as point color and shape.

A Principal Component Analysis was performed to further explore possible relations among examined traits (**Figure 4**). In general, the distribution of texture parameter followed that previously reported by Crespan et al. (2021): berry hardness-derived parameters (BH, BG, and BCh, all expressed in N) are positively correlated mostly to Dim 1 (53.1% of variance explained), while the dimensionless parameters BCo, BS_ratio, and BR negatively correlated to them. Berry skin breakage traits (F_sk_, W_sk_, and E_sk_) are mostly represented by Dim 2 (16.6% of explained variance; loadings F_sk_ -0.354, W_sk_ -0.298, E_sk_ -0.391), although closer to the whole berry hardness-derived parameters with respect to previous evaluations. The berry skin thickness (Sp_sk_) confirms its lack of substantial correlation with other not-normalized texture parameters, as previously assessed with Pearson’s correlation coefficients (**Figure 3**). For seeds traits, all parameters show general correlation among them, being plotted in the same direction prominently following Dim 1, with a limited exception for S parameter. Finally, considering the interaction among seeds and berry texture data, it emerges that whole berry hardness-derived parameters and most seeds parameters are positively correlated with Dim 1, reinforcing the pairwise correlations reported with Pearson’s coefficients (**Figure 3**).

### 3.2 Map construction

The grapevine SPET pipeline applied in this study led to the identification of a total of 16, 026 loci carrying a genome coverage higher than 10x. Of these, 7, 558 were polymorphic in at least one parent. After the filtering for MAF > 10%, binning, and SD analysis, 2, 904 (59.64% of total filtered no SD loci) and 2, 890 (59.36% of total filtered no SD loci) polymorphic markers were identified for Raboso Veronese (RabVe) and Sultanina (Sul), respectively. These markers were categorized into five groups based on their segregation pattern in the two parents: ab × aa or ab × cc (1:1 segregation only in RabVe; 1, 956 and 23, respectively), aa × ab or cc × ab (1:1 segregation only in Sul; 1, 955 and 10, respectively), ab × ab (1:2:1 segregation with the same alleles in both parents; 850 in total), ab × cd (1:1:1:1 segregation with different alleles in the two parents; 38 in total), and ab × ac (1:2:1 segregation with one common allele in both parents; 37 in total). The 850 ab × ab markers were excluded from the pseudo-test cross map construction as it was not possible to determine the phase in the heterozygous individuals of the progeny. Finally, 1, 223 markers (42.11% of Raboso Veronese polymorphic filtered markers) were mapped for Raboso Veronese, while 1, 238 markers (42.84% of Sultanina polymorphic filtered markers) were anchored for Sultanina. **Table 3** summarizes the total numbers of markers obtained after each step of the analysis.

**Table 3:** Summary of markers obtained after each step of filtering, classification, and mapping.

Total	Filtering for MAF > 10%	Binning	no SD markers	Polymorphic markers		Mapped markers	
				Raboso Veronese	Sultanina	Raboso Veronese	Sultanina
7558 (40 ab x cd 44 ab x ac 1806 ab x ab 2791 ab x aa 26 ab x cc 2841 aa x ab 10 cc x ab)	7186 (95.08% of total) (38 ab x cd 37 ab x ac 1796 ab x ab 2613 ab x aa 24 ab x cc 2668 aa x ab 10 cc x ab)	6375 (88.71% of filtered for MAF>10%) (38 ab x cd 37 ab x ac 1617 ab x ab 2277 ab x aa 23 ab x cc 2373 aa x ab 10 cc x ab)	4869 (76.38% of filtered for bins)	2904 (59.64% of no SD markers) (38 ab x cd 37 ab x ac 850 ab x ab 1956 ab x aa 23 ab x cc)	2890 (59.36% of no SD markers) (38 ab x cd 37 ab x ac 850 ab x ab 1955 aa x ab 10 cc x ab)	1223 ab x aa (42.11% of Raboso Veronese polymorphic markers)	1238 aa x ab (42.84% of Sultanina polymorphic markers)

The first allele in the segregation pattern is referred to Raboso Veronese, while the second one corresponds to Sultanina. MAF: Minimum Allele Frequency; SD: Segregation Distortion.

All the mapped loci displayed a 1:1 segregation type (**Table 3**) and were represented by SNPs, except for an InDel at locus chr18_27298764 on Sultanina chromosome 18.

**Figure 5:**
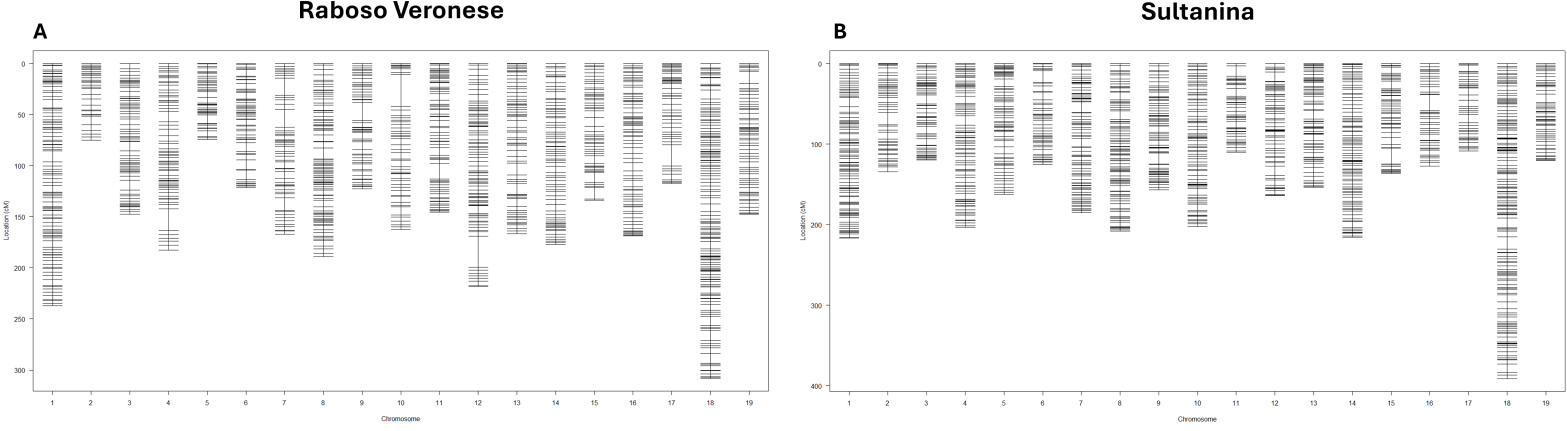
Graphical representations of the genetic maps obtained for the two parents Raboso Veronese and Sultanina. **(A)** Distribution of the genetic position (cM) of markers across each LG for Raboso Veronese. Each bar indicates a single locus. **(B)** Distribution of the genetic position (cM) of markers across each LG for Sultanina. Each bar indicates a single locus.

In the pseudo-test cross linkage mapping, 19 LGs, corresponding to the 19 V*. vinifera* chromosomes, were obtained for both genotypes (**Figure 5**; **Supplementary Files 1**, **2**).

For Raboso Veronese, markers were distributed over 3, 062.44 cM, with a genome-wide average density of 0.40marker/cM. For Sultanina, markers were anchored on a total length of 3, 242.02 cM, with a mean density of 0.38 marker/cM (**Table 4**).

**Table 4:** Summary of data obtained for each LG in the Raboso Veronese and Sultanina corresponding genetic maps.

LG	No. of markers		Lenght (cM)		Density (marker/cM)		Correlation between genetic and physical positions	
	Raboso Veronese	Sultanina	Raboso Veronese	Sultanina	Raboso Veronese	Sultanina	Raboso Veronese	Sultanina
1	96	89	237.03	216.59	0.41	0.41	0.98	0.99
2	30	48	75.10	134.24	0.40	0.36	0.98	0.96
3	64	52	147.46	119.8	0.43	0.43	0.86	0.88
4	66	77	182.81	203.38	0.36	0.38	0.97	0.98
5	40	63	74.49	162.5	0.54	0.48	0.99	0.97
6	52	44	121.45	125.20	0.43	0.35	0.99	0.99
7	51	76	166.98	185.08	0.38	0.41	0.99	0.98
8	84	85	189.23	207.98	0.45	0.41	0.98	0.97
9	50	64	122.56	156.93	0.41	0.41	0.97	0.93
10	47	75	162.14	202.38	0.29	0.37	0.96	0.82
11	65	42	145.66	110.22	0.45	0.38	0.96	0.97
12	83	57	218.11	163.97	0.38	0.35	0.94	0.95
13	61	64	166.71	153.93	0.36	0.42	0.99	0.97
14	71	85	177.09	215.53	0.40	0.39	0.97	0.98
15	53	49	133.81	136.75	0.40	0.36	0.98	0.91
16	70	39	168.75	127.19	0.41	0.31	0.97	0.97
17	45	37	117.50	108.77	0.38	0.34	0.98	0.99
18	135	141	308.06	391.16	0.44	0.36	0.96	0.98
19	60	51	147.50	120.42	0.41	0.42	0.95	0.96
**Total**	**1223 SNPs**	**1238**	**3062.44**	**3242.02**	**M 0.40**	**M 0.38**	**M 0.97**	**M 0.96**

For each LG in the two genotypes the total number of mapped markers, genetic length, marker density, and Spearman correlation index between genetic positions and physical positions, annotated on *V. vinifera* 12x.v0 genome (http://vitisgdb.ynau.edu.cn/downloads.html), of markers are indicated. For both maps, total number of markers, total genetic length, and mean marker density are also shown. M, Mean.

The number of mapped markers and their density in the two genetic maps were comparable, Sultanina map being 179.58 cM longer than that of Raboso Veronese.

LG18 was the longest and contained the highest number of markers in both parents, while LG2 in Raboso Veronese and LG17 in Sultanina had the least markers. The highest density was observed on LG5 in both genotypes (0.54 marker/cM in Raboso Veronese and 0.48 marker/cM in Sultanina), whereas the lowest ones were detected for Raboso Veronese LG13 and Sultanina LG17 (**Table 4**).

Large gaps, spanning more than 20 cM, were present mainly in Raboso Veronese LG4 (from chr4_20120842 to chr4_22278419), LG10 (from chr10_722768 to chr10_1430111), LG12 (from chr18_2669811 to chr12_21019751), and LG17 (from chr17_7940429 to chr17_10713842) (**Figure 5A**, **Supplementary File 2**).

For each LG in the two maps, the genetic positions of the markers were correlated to the physical positions annotated on the grape reference genome 12x.v0 (http://vitisgdb.ynau.edu.cn/downloads.html) and high levels of genetic collinearity were found, with correlation mean values of 0.97 and 0.96, respectively for Raboso Veronese and Sultanina (**Table 4**; **Supplementary File 1**). Nonetheless, rearrangements in comparison to reference genomic sequence occurred. An inversion was identified on LG3 for both genotypes: from chr3_5739074 to chr3_3575944 in Raboso Veronese and from chr3_5528953 to chr3_4283297 in Sultanina. Inversions occurred also on Raboso Veronese LG10 (from chr10_15397539 to chr10_12745934) and on Sultanina LG15 (from chr15_20182161 to chr15_18892517) (**Supplementary Files 1**, **2**).

In addition, some markers were mapped on different chromosomes in comparison to the annotated ones; chr18_2669811, annotated on LG18 on the reference genome, was mapped at 199.5 cM on Raboso Veronese LG12; chr11_15815289 and chr11_15815326, annotated on LG11, were located, respectively, at 48.39 and 48.37 cM on Sultanina LG8.

### 3.3 Identification of QTLs associated with berry and seed traits

**Figure 6:**
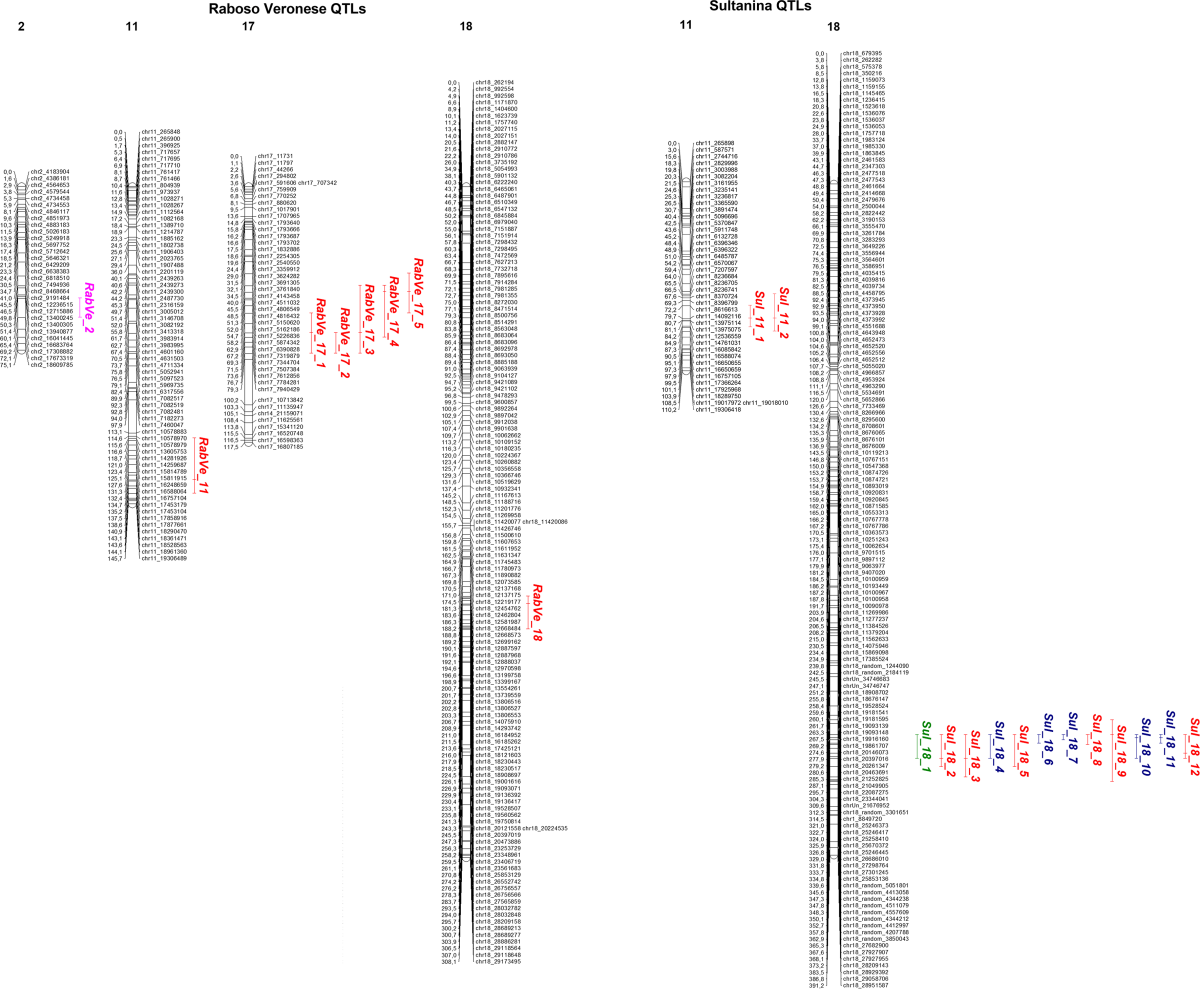
Graphical representation of the QTLs identified for each parent. The LGs in which QTLs have been detected are reported as graphic bars. Each LG number is indicated above the map bars. For each association, QTL acronym and the corresponding CI are indicated. CIs are represented as color bars that include the peak marker as a dash. Berry color related QTL is in purple, berry specific QTLs are in red, seed specific QTLs are in blue, while the QTL related to both berry and seed traits is indicated in green. Peak markers are indicated by bars inside the QTL intervals when they do not correspond to flanking markers.

A total of 22 genomic regions significantly co-segregating with berry texture and/or seed traits were identified across both parental maps (**Figure 6**).

These corresponded to 10 distinct peak markers and comprised 8 and 14 QTLs in Raboso Veronese and Sultanina, respectively. QTLs located on the same LG within the same genetic background showed overlapping CIs, while no epistatic interactions were detected between loci controlling the same trait. Each association was named using an acronym indicating the parental map (‘RabVe’ for Raboso Veronese, ‘Sul’ for Sultanina), the corresponding LG number, and, when multiple QTLs occurred on the same LG, a sequential identifier. Summary information for the main associations is provided in **Table 5**, while complete results are reported in **Supplementary File 3**. Overall, QTLs were detected for 29 out of the 33 considered traits, excluding BCh_sur, BG_sur, F_sk_, and S.

**Table 5:** Main results related to the QTLs identified in the two parents, Raboso Veronese and Sultanina.

QTL acronym	Peak marker	CI (cM)	Trait	CC	LOD	EPV (%)	a
*RabVe_2*	chr2_16041445	51.4 - 60.06	BC	nd	54.88	64.18	0.002
*RabVe_11*	chr11_17453179	115.6 - 140.89	E_sk_	2	4.75	10.23	0.029
*RabVe_17_1*	chr17_7319879	58.24 - 76.71	BH	1_2	4.8	11.16	0.85
*RabVe_17_2*		67.23 – 76.71	BG	1_2	3.89	7.53	0.405
			BCh		3.65	7.5	0.315
			BG_diam	1_1	5.56	11.05	0.221
			BCh_diam		5.11	9.96	0.167
*RabVe_17_3*		45.47 - 76.71	BH_diam		6.77	13.77	0.482
			BH_sur	4	5.93	13.5	0.176
*RabVe_17_4*	chr17_4816432	45.47 – 69.24	BR	8	4.13	10.56	0.018
*RabVe_17_5*		40.04 – 58.24	BCo		5.31	12.13	0.029
*RabVe_18*	chr18_12887968	188.22 – 203.34	W_sk_	2	4.19	9.74	0.093
*Sul_11_1*	chr11_14092116	65.5 – 87.3	BG	1_2	5.08	12.5	-0.505
*Sul_11_2*		65.5 – 103.93	BCh	1_2	5.26	12.85	-0.412
*Sul_18_1*	chr18_25853136	320.97 – 334.82	NS	3	45	65.49	39.993
			DSW		32.88	56.29	1.157
			ABW		23.3	46.02	1.153
*Sul_18_2*		320.97 – 339.61	BH	1_2	18.32	36.13	1.511
			BG		19.9	36.62	0.851
			BCh		19.78	37.3	0.681
			BG_vol	5	12.91	29.64	-0.552
			BCh_vol		13.76	32.54	-0.483
*Sul_18_3*		320.97 – 345.62	BH_vol	5	9.27	23.42	-0.741
*Sul_18_4*	chr18_25246373	320.97 – 334.82	FSW	3	23.61	47.12	1.590
			FSW/DSW	7	26.4	51.05	-1.236
			FeretAR		26.8	44.52	-0.523
*Sul_18_5*		320.97 – 339.61	BH_diam	1_1	8.37	17.71	0.575
			BG_diam		7.51	15.78	0.277
			BCh_diam		6.66	14.21	0.206
			BS_ratio	8	5.12	10.91	-0.012
*Sul_18_6*		320.97 – 325.88	Circ	3	18	32.27	0.107
*Sul_18_7*		320.97 – 323.98	R	3	27.5	45.36	0.148
*Sul_18_8*		320.97 – 326.8	BCo	8	4.24	9.24	-0.026
*Sul_18_9*		312.25 – 348.33	BR	8	5.91	13.95	-0.021
*Sul_18_10*	chr18_25246417	320.97 – 334.82	A	3	38.57	59.42	9.221
			P		27.7	47.13	5.065
			F		24.3	43.39	1.642
*Sul_18_11*		320.97 – 325.88	MF	3	37.2	60.31	1.532
*Sul_18_12*	chr18_27298764	320.97 – 334.82	Sp_sk_	nd	4.58	8.06	21.831

For each QTL, the corresponding acronym, peak marker, confidence interval (CI) on the genetic map, associated trait/s, with the corresponding Correlation Cluster (CC), LOD, explained phenotypic variance (EPV), and additive effect (a) are reported. QTL acronyms consist of three pieces of information, separated by an underscore: the parental map in which it was found (‘RabVe’ for Raboso Veronese map and ‘Sul’ for Sultanina map), the number of the corresponding LG, and, in case more than one QTL mapped on the same LG, a sequential number. Full results are shown in **Supplementary File 4**; nd, not determined.

In Raboso Veronese, the most significant association corresponded to a major QTL for berry color (*RabVe_2*) on LG2, explaining 64.18% of the phenotypic variance (EPV) and with a high LOD score of 54.88. Loci associated with berry texture were identified on LGs 11, 17, and 18. In particular, LG17 harbored two groups of QTLs including, respectively, three and two minor QTLs associated with texture parameters, each defined by a distinct peak marker. Two TPA-related loci were specifically linked to skin-associated traits (E_sk_ and W_sk_). No seed-related QTLs were found in this parent.

In Sultanina, 14 QTLs co-segregating with berry texture and/or seed traits were detected in LGs 11 and 18. In more detail, two QTLs on LG11 (*Sul_11_1* and *Sul_11_2*) were associated with BG and BCh, respectively, and shared the same peak marker. Notably, this region was located near the E_sk_-associated locus *RabVe_11* in Raboso Veronese, suggesting the presence of a conserved genomic region on chromosome 11 influencing berry traits in both parental backgrounds. However, marker density in this region was relatively low, with gaps of approximately 5.5 Mb in Sultanina and 3 Mb in Raboso Veronese (**Supplementary File 2**), indicating that additional markers may be required to refine the CI boundaries.

The most complex QTL pattern was observed on Sultanina LG18, which harbored 12 loci associated with berry texture and/or seed traits grouped around four closely spaced peak markers. These loci explained a substantial proportion of EPV (up to 65.49%), with LOD values up to 45, and were associated with multiple correlated traits, including seed size parameters and berry texture variables. The clustering of highly correlated traits within the same genomic regions suggests pleiotropic effects rather than tight linkage, a hypothesis further supported by multi-trait QTL analyses.

Overall, these results highlighted several genomic regions controlling berry texture and/or seed-related parameters in the two parents, with the major hotspot located on Sultanina LG18 and additional loci identified on LG11, in both genotypes, and LG17, in Raboso Veronese.

In the present study, QTL mapping was carried out independently on parental maps constructed using a pseudo-test cross. Consequently, for each QTL the resulting additive effect points out the effect of inheriting one allele *versus* the alternative allele from the heterozygous parent, that is each of these effects indicate a within-parent allelic substitution effect. The analysis of additive effects of QTL detected for each trait showed two Sultanina QTLs (*Sul_11_2* and *Sul_18_2*) co-segregating with BCh with contrasting additive effects (**Table 5**). The same pattern was detected for BCo_diam, BCo_sur and BG traits, as Sultanina QTLs co-segregating with each of these traits showed contrasting additive effects (**Table 5**). Interestingly, our analysis detected pleiotropic QTLs (e.g. Sul_18_1) showing contrasting additive effects on different traits which might be arisen from trait correlations or multiple tightly linked genes, each of which affecting traits in different directions (**Table 5**). Finally, the large additive effect of the QTL co-segregating with NS reflects the analyses carried out for this trait, which was measured on a set of 25 berries; that of the QTL co-segregating with Sp_sk_ reflects the low measure unit used (μm).

### 3.4 Consistent and novel QTLs reveal the genetic basis of berry and seed development status

Literature searches highlighted correspondences between several loci mapped in this study and previously reported regions associated with berry color and texture.

The major BC-associated QTL *RabVe_2* co-localized with the BC-related locus *col_2_1*, identified in an F_1_ population derived from the cross Red Globe × Muscat Hamburg (Sun et al., 2020), and overlapped with an approximately 8.4-Mb interval on chromosome 2 associated with BC in a SPET-based GWAS of 84 Croatian grapevine accessions (Marinov et al., 2024) (**Table 6**), corroborating the pipeline of QTL analyses used in the present study.

**Table 6:** Correspondence between QTLs identified in this study and previously reported loci related to berry traits in grapevine.

QTL(s) (this study)	Locus(i)/QTL(s) (previous studies)	Genetic material Mapping method	Genomic location (bp) Reference genome	Ref.
*RabVe_2*	*col_2_1*	Red Globe × Muscat Hamburg F_1_ (95 individuals); WGS-based linkage mapping	13368581 – 16199608 (PN40024 12x.v0)	Sun et al., 2020
	BC-associated region on chr 2	84 Croatian grapevine accessions; SPET-based GWAS	7159705 – 18424248 (PN40024 12x.v0)	Marinov et al., 2024
*RabVe_17_3, RabVe_17_4, RabVe_17_5*	Marker VMC3A9	Raboso Veronese × Sultanina F_1_ (152 individuals); SSR-based linkage map	4984101 (PN40024 12x.v0)	Crespan et al., 2021
*Sul_11_1, Sul_11_2, RabVe_11*	*MesF11.1, MesF11.3, PPH11.2, PPH11.3*	Red Globe × Muscat Hamburg F_1_ (151 individuals); WGS-based linkage mapping	12498489 – 15204246 13466880 – 15168179 12451342 – 13460540 12439382 – 15204246 (PN40024 12x.v2)	Lin et al., 2023
*Sul_18* QTLs	*qBF18-2016, qBF18-2017, qBF18-2018*	Muscat Hamburg × Crimson Seedless F_1_ (105 individuals); WGS-based linkage mapping	26582255 – 27199073 25029102 – 26343343 25053661 – 27380155 (PN40024 12x.v0)	Jiang et al., 2020
	Marker AGL11-VMC7F2	Raboso Veronese × Sultanina F_1_ (152 individuals); SSR-based linkage map	26896790 (PN40024 12x.v0)	Crespan et al., 2021
		Muscat Hamburg × Sugraone F_1_ (153 individuals); Ruby Seedless × Moscatuel F_1_ (78 individuals); SSR-based linkage mapping		Carreño et al., 2015

For each correspondence the QTL/QTLs identified in the present study, the locus/loci or QTL/QTLs reported in previous studies, with the corresponding genetic material, mapping method, genomic location, genome reference, and literature reference, are indicated.

Consistent with our findings, previous studies reported associations between berry texture and loci on chromosome 17. In an SSR-based linkage mapping study using the same F_1_ population examined in the present study, Crespan et al. (2021) linked the SSR marker VMC3A9 to several texture traits, including BR, BCo, BH_diam, and BG_diam. This marker, located at 4, 984, 101 bp on chromosome 17, falls within the CIs of *RabVe_17_3*, *RabVe_17_4*, and *RabVe_17_5*, and is positioned only 167, 669 bp downstream of the *RabVe_17_4* and *RabVe_17_5* peak marker chr17_4816432 (**Table 6**). This data strongly suggests that VMC3A9 and the QTLs identified on Raboso Veronese LG17 likely represent the same genetic region controlling berry texture.

Further evidence of correspondence with previously characterized loci was found on chromosome 11. The Red Globe × Muscat Hamburg cross mentioned above was used to develop an F_1_ population of 151 individuals analyzed by whole-genome sequencing (Lin et al., 2023). In that study, molecular markers were distributed into blocks and 27 loci, across 11 chromosomes, were associated with mesocarp firmness (MesF), pericarp puncture hardness (PPH), and pericarp brittleness (PerB). Four QTLs on chromosome 11, *MesF11.1*, *MesF11.3*, *PPH11.2*, and *PPH11.3*, showed CIs included in the berry-related *RabVe_11*, *Sul_11_1*, and *Sul_11_2* identified in the present study (**Table 6**, **Supplementary File 3**). Moreover, the peak marker chr11_14092116 of both *Sul_11_1* and *Sul_11_2* lies within the peak block of *PPH11.3* (Block740, 13, 466, 880–14, 301, 707 bp) (Lin et al., 2023), supporting the hypothesis that the genomic region of two Sultanina LG11 QTLs might correspond to the *PPH11.3* locus.

The 12 QTLs on Sultanina LG18 (**Supplementary File 3**) were in close proximity of three previously reported QTLs for berry firmness (*qBF18-2016*, *qBF18-2017*, and *qBF18-2018*) (**Table 6**), identified through WGS-based linkage mapping in a Muscat Hamburg × Crimson Seedless F_1_ population of 105 individuals (Jiang et al., 2020). These three loci explained over 20% of the phenotypic variance and were consistently detected across three growing seasons. Notably, the peak marker of the Sp_sk_ *Sul_18_12* (chr18_27298764) was located only 99, 691 bp downstream of the *qBF18–2016* peak marker (27, 199, 073 bp) (Jiang et al., 2020) (**Table 6**, **Supplementary File 3**).Moreover, the Sultanina LG18 QTL cluster also coincides with a major berry texture locus previously reported by Crespan et al. (2021), where the microsatellite AGL11-VMC7F2, annotated at 26, 896, 790 bp on chromosome 18, inside the promoter region of the *VviAGL11* gene, was identified as the peak marker. The same marker was also associated with berry firmness in two independent *V. vinifera* segregating populations derived from Muscat Hamburg × Sugraone (153 progeny) and Ruby Seedless × Moscatuel (78 progeny) (Carreño et al., 2015) (**Table 6**).

Altogether, these overlaps across independent mapping populations and methodologies confirm the robustness of the present QTL detections and reinforce the role of the identified regions on chromosomes 2, 11, 17, and 18 as key genomic determinants of berry color, seed development status and berry texture in grapevine.

On the other hand, the W_sk_ locus *RabVe_18*, associated with W_sk_, did not correspond to any previously reported berry texture QTL. Similarly, the associations identified on chromosome 18 in Sultanina for seed development status–related traits have not been described before, indicating that these loci may represent novel genomic regions controlling these traits. Nevertheless, their co-localization with the QTL linked to the AGL11–VMC7F2 marker, and thus with *VviAGL11*, a major regulator of seedlessness (Wang et al., 2024) (see below), supports the biological relevance of these findings and suggests that a common region on chromosome 18 contributes to both seed development status and berry texture.

### 3.5 Analysis of annotated genes located inside the confidence intervals of the detected QTLs

The loci within the CIs of the QTLs detected in this work, according to the *V. vinifera* reference genome annotation (PN40024 12x.v5), were examined to identify genes with putative roles in the genetic regulation of berry texture and/or seed development status (**Supplementary File 4**). Because the physical positions on the grape genome of markers chr18_random_4413058 (right flanking marker of *Sul_18_2*), chr18_random_5051801 (right flanking marker of *Sul_18_3*, *Sul_18_4*, and *Sul_18_6*), and the *Sul_18_10* flanking markers chr18_random_3301651 and chr18_random_4557609 were unknown (**Supplementary File 3**), the positions of the nearest markers located within the corresponding CIs were used to define the intervals for annotated gene search (**Supplementary File 2**).

All the identified CIs contained a large number of annotated genes. Specifically, 120 genes (93 of which were functionally classified) were identified for *RabVe_2*; 583 genes (471 functionally classified) were located within the QTLs on chromosome 11; and 357 genes (318 functionally classified) were found within Raboso Veronese QTLs on chromosome 17. Additionally, 105 genes (92 functionally classified) were detected for *RabVe_18*, whereas a total of 169 genes (147 functionally classified) were annotated within Sultanina QTLs on chromosome 18 (**Supplementary File 5**).

As expected, the cluster of *VviMYBA* genes (*VviMYBA1*, *VviMYBA2*, and *VviMYBA3*), whose combined activity determines berry color in grapevine (Fournier-Level et al., 2009), was located within the *RabVe_2* CI (**Table 7**, **Supplementary Files 3**, **5**).

**Table 7:** Summary of the genes carrying function ascribable to roles in the determination of berry texture and/or seed development status inside the CI of each QTL detected in the present study.

Trait/s	QTL/s	ID	Description	Putative biological process	Ref.
BC	*RabVe_2*	Vitvi02g01257	*VviMYBA2*	Anthocyanin biosynthesis	Walker et al., 2007; Fournier-Level et al., 2009; Bao et al., 2025
		Vitvi02g01260	*VviMybA1*		
		Vitvi02g01265	*VviMYBA3*		
Berry texture	*RabVe_17_1, 2*, and *3*	Vitis17g00828	UDP-glucosyltransferase	Cell wall polysaccharide remodeling	Asif et al., 2014; Hossain et al., 2014; Chandrasekar and Van Der Hoorn, 2016; Amos and Mohnen, 2019; Hrmova et al., 2022; Zhong et al., 2024
		Vitis17g00829			
		Vitis17g00835			
Berry texture	*RabVe_17_1, 3*, and *4*	Vitis17g00773	Glycosyltransferase	Cell wall polysaccharide remodeling	
Berry texture	*RabVe_17_3, 4*, and *5*	Vitis17g00551	UDP-glucosyltransferase	Cell wall polysaccharide remodeling	
		Vitis17g00552			
		Vitis17g00575	Glycosyltransferase		
		Vitis17g00577			
Berry texture	*RabVe_17_5*	Vitis17g00493 Vitis17g00495 Vitis17g00499		Cell wall signaling and integrity sensing	Seifert and Blaukopf, 2010; Kohorn and Kohorn, 2012
			WAK		
Berry texture	*RabVe_18*	Vitis18g01304	GDSL lipase/esterase	Cuticle remodeling and cell wall modification	Zhang et al., 2017; Ni et al., 2020
		Vitis18g01379	Glycosyltransferase 8	Cell wall polysaccharide synthesis	Asif et al., 2014; Hossain et al., 2014; Chandrasekar and Van Der Hoorn, 2016; Amos and Mohnen, 2019; Hrmova et al., 2022; Zhong et al., 2024
		Vitis18g01357	Sugar transporter SWEET	Sugar transport and partitioning	Zhang et al., 2019; Ren et al., 2020
Berry texture	*RabVe_11*, *Sul_11_1* and *2*	Vitis11g01033	Glycosyltransferase	Cell wall polysaccharide remodeling	Asif et al., 2014; Hossain et al., 2014; Chandrasekar and Van Der Hoorn, 2016; Amos and Mohnen, 2019; Hrmova et al., 2022; Zhong et al., 2024
		Vitis11g01035	SBP domain	Fruit development	Cui et al., 2018
Berry texture	*RabVe_11, Sul_11_2*	Vitis11g01296	Glycosyltransferase	Cell wall polysaccharide remodeling	Asif et al., 2014; Hossain et al., 2014; Chandrasekar and Van Der Hoorn, 2016; Amos and Mohnen, 2019; Hrmova et al., 2022; Zhong et al., 2024
Berry texture	*Sul_11_1* and *2*	Vitis11g00949	FAS1 fasciclin-like arabinogalactan protein	Cell wall organization	Lin et al., 2023
Berry texture	*Sul_18_1, 2, 3, 4, 5, 9, 10*, and *12*	Vitis18g02646	GDSL lipase/esterase	Cuticle remodeling, cell wall modification	Zhang et al., 2017; Ni et al., 2020
Berry texture/Seed development status		Vitis18g02523	Chaperone DnaK	Protein folding, hormone signaling	Pilati et al., 2014; Zhang et al., 2008; Sung et al., 2001; Lin et al., 2023
		Vitis18g02617	*VviAGL11*	Seed coat development, ovule identity	Smaczniak et al., 2012; Shah et al., 2022; Crespan et al., 2021
		Vitis18g02644	*VviAGL12*		Mao et al., 2023
		Vitis18g02628	*OPR11_1*	Jasmonate biosynthesis and developmental regulation	Olmedo et al., 2023
		Vitis18g02630	*OPR2_4*		
		Vitis18g02638	*OPR2_7*		

For each gene, the corresponding trait, QTL/s, ID, and functional description with documented biological process/es are indicated.

For the other QTLs identified in both genotypes, two peak markers co-localized with genes annotated on the grapevine reference genome with functions potentially ascribable to the regulation of berry texture or seed development status.

For example, chr18_25853136, the peak marker of the berry and seed-associated QTL *Sul_18_1* and of the berry-related QTLs *Sul_18_2* and *Sul_18_3*, was positioned within the eighth exon of Vitis18g02523, encoding a DnaK (Hsp70-like) chaperone (**Table 7**; **Supplementary File 5**). The corresponding C/A substitution results in a Pro677Thr amino acid change in the protein sequence (**Supplementary File 6**). Hsp70 chaperones are widely expressed during fruit development and ripening (Pilati et al., 2014; Zhang et al., 2008) and have been shown to contribute to seed development and size regulation (Sung et al., 2001; Zhang et al., 2023). Furthermore, the *Sul_18_12* peak chr18_27298764 was located within the 5′ UTR of Vitis18g02646, encoding a GDSL lipase/esterase (**Table 7**, **Supplementary File 5**). This enzyme class is involved in the deacetylation of cell wall polysaccharides during early fruit development and is transcriptionally regulated throughout ripening in grapevine (Zhang et al., 2017; Ni et al., 2020). On this basis, Vitis18g02523 and Vitis18g02646 can be considered targets for future investigations aimed at defining their potential roles in the phenotypic effects associated with the *Sul_18* QTLs.

Interestingly, within the CIs of most Sultanina LG18 QTLs, (*Sul_18_1*, *2*, *3*, *4*, *5*, *9*, *10*, and *12*) two MADS-box AGAMOUS-like genes, *VviAGL11* and *VviAGL12*, were consistently detected (**Table 7**, **Supplementary File 5**). *VviAGL11* encodes a transcription factor regulating floral organ and fruit development, as well as seed size in grapevine (Sreekantan et al., 2006; Smaczniak et al., 2012; Wang et al., 2021; Amato et al., 2022; Shah et al., 2022). *VviAGL12* was introduced and overexpressed into an *Arabidopsis* ecotype mutant for its homologue gene. The resulting lines showed improved fresh weight, root length, plant height, and seed production, as well as reduced flowering time (Mao et al., 2023). In the same genomic region of *VviAGL11* and *VviAGL12*, a cluster of NADH:flavin oxidoreductase/NADH oxidase genes, Vitis18g02628 (*OPR11_1*), Vitis18g02630 (*OPR2_4*), and Vitis18g02638 (*OPR2_7*), encode putative 12-oxophytodienoate reductases (**Table 7**, **Supplementary File 5**), enzymes involved in jasmonate biosynthesis and, consequently, in fruit development and texture modulation (Olmedo et al., 2023).

Other genes, listed in **Table 7**, with potential roles in berry and seed development status are further examined in the Discussion section.

Overall, all these observations revealed the presence of genes with annotated functions compatible with a role in the definition of the traits considered in this study inside the CIs of all the detected QTLs.

## 4 Discussion

### 4.1 SPET-based genotyping and linkage mapping

In the present work, the first SPET-based high resolution pseudo-test cross linkage maps were developed starting from a grapevine F_1_ populationAlthough SPET allowed detecting different types of polymorphic co-dominant markers, the final linkage maps showed in the present study include only SNPs in Raboso Veronese and SNPs plus only one InDel in Sultanina, totalling 1, 223 and 1, 238 markers per map, respectively.

The suitability of SPET genotyping for linkage map construction in highly heterozygous species like *V. vinifera* can be biased by allele miscalling and missing data, resulting in erroneous SNP detection (Barba et al., 2014; Cadle-Davidson et al., 2016; Possamai et al., 2021) and leading to cumulative overestimation of recombination frequencies between closely linked markers (Scheben et al., 2020; Furuta et al., 2017; Bajgain et al., 2016).

Map expansion can result from high heterozygosity, particularly when linkage phase is unresolved. In pea, F_2_ maps were longer despite the consistent marker order across LGs, indicating that heterozygosity mainly inflates recombination estimates without altering map structure (Knox and Ellis, 2002).

In our case, a filtering threshold of 6 reads led to the development of extremely inflated maps (~15, 000 cM) (data available on request), a length 10 times exceeding those reported in previous *Vitis* linkage maps, typically ranging from ~1, 000 to ~1, 500 cM (Dvořák et al., 2025; Frenzke et al., 2024; Lin et al., 2023; Shi et al., 2022; Possamai et al., 2021; Su et al., 2021; Jiang et al., 2020; Hyma et al., 2015). This result confirms that even small genotyping inaccuracies can dramatically influence total map length. The application of a higher filtering threshold (usage of at least 10 reads to support allele call) resulted in a total length of about 3, 000 cM, with mean inter-marker distances below 3 cM for both parents, suggesting that although stringent filtering, binning, and ordering procedures were applied, minor inconsistences likely persisted, contributing to moderate map inflation. The use of a bi-parental F_1_ population, with partially unresolved linkage phase, may have further complicated the recombination estimation.

Structural discrepancies relative to the reference genome were also observed, including chromosomal inversions (on chromosome 3 in both parents and on chromosome 10 and 15 in Raboso Veronese and Sultanina, respectively) and misassigned markers, likely reflecting chromosomal rearrangements, genotyping errors, or local mis-assemblies in the reference Pinot noir genome.

We acknowledge that the sequencing coverage could be increased to further reduce errors in recombination estimates. Alternatively, genotyping in technical replicates or integrating independent datasets could help to reduce random errors and to improve SNP calling accuracy. Nevertheless, the strong correlation observed between genetic and physical positions confirms that marker ordering was accurate and recombination estimates, while slightly inflated, remained biologically consistent. Despite these limitations, the linkage maps developed here provide a valuable genomic resource for grapevine, supporting QTL detection and refinement of the *V. vinifera* genome assembly.

### 4.2 Comparison with QTLs found in previous studies and updating

As indicated in Results, the robustness and reliability of our experimental pipeline are supported by the concordance between several QTLs detected in this study and previously reported loci co-segregating with berry color or texture. However, some discrepancies were also observed. For instance, we examined the colocalization of the QTLs on Sultanina LG18 with the three berry firmness–associated regions (*qBF18-2016*, *qBF18-2017*, and *qBF18-2018*) reported by Jiang et al. (2020). In that work, structural rearrangements on chromosome 18, relative to the reference genome, were detected. In contrast, our genetic map displayed a high degree of collinearity between genetic and physical marker positions within the CIs of Sultanina LG18 QTLs, with no evidence of major rearrangements. Only the marker chr18_25853136 was positioned after chr18_27301245 (**Supplementary File 2**). Moreover, we successfully mapped 9 markers, annotated as randomly assigned to chromosome 18, between chr18_25853136 (334.82 cM) and chr18_27682900 (365.53 cM) (**Supplementary File 2**).

The discrepancies with earlier reports may therefore reflect differences in parental genotypes, mapping strategies, or marker resolution. Considering that structural variation in grapevine is often genotype-specific (Cardone et al., 2016; Djari et al., 2024), such inconsistencies likely mirror the natural structural diversity within the species. Taken together, these findings suggest that no rearrangements were detected within the mapped regions on chromosome 18 in Sultanina compared to the reference genome. This structural stability may have facilitated the co-localization of multiple QTLs influencing correlated berry and seed traits, supporting the hypothesis of a shared genetic basis and pleiotropic effects within this genomic region. The high LOD scores observed further indicate strong genetic effects, suggesting that these loci may act as key regulators driving a coordinated genetic modulation of berry characteristics and seed development failure.

Our study also identified novel associations related to the traits considered, including *RabVe_18* locus linked to W_sk_, and the seed-related QTLs on Sultanina chromosome 18. Collectively, this evidence not only highlights the power of our linkage mapping approach to uncover novel genomic associations underlying the investigated traits but also expands our understanding of the complex genetic architecture underlying berry texture and seed defective development in grapevine. Our results provide new loci that may serve as promising targets for breeding programs aimed at fine-tuning berry texture and seed traits in cultivated varieties.

### 4.3 *In silico* functional analysis of genes annotated into QTL regions associated with berry and seed traits

*In silico* analysis of genes annotated inside the Cis of the detected QTLs enabled the identification of genes with functions potentially related to berry texture and seed development status determination in the two grapevine genotypes.

Multiple glycosyltransferases and UDP-glucosyltransferases map within the CIs of Raboso Veronese QTLs on LGs 17, 11, and 18 (**Table 7**, **Supplementary File 5**). These enzymes belong to families that catalyze the formation of linkages in cell wall matrix polysaccharides, contributing to the complex changes in cell size, shape, mechanical strength, and adhesion that occur during plant development and have been depicted as targets for genetic manipulation for desirable traits of fruits (Asif et al., 2014; Hossain et al., 2014; Chandrasekar and Van Der Hoorn, 2016; Amos and Mohnen, 2019; Hrmova et al., 2022; Zhong et al., 2024). Since cell wall composition is a key determinant of fruit tissue firmness and softness in grapevine (Hu et al., 2025), the presence of glycosyltransferase genes within Raboso Veronese loci associated with berry texture represents a valuable starting point for future studies aimed at exploring their impact on grape berry mechanical properties. Besides glycosyltransferases, within *RabVe_18*, a GDSL lipase/esterase (Vitis18g01304) was also identified. GDSL lipases, involved in deacetylation of cell wall polysaccharides and biosynthesis of cutin and wax, can affect the elasticity and integrity of the fruit skin and thus fruit tissue firmness and softness (de la Torre et al., 2002; Zhang et al., 2017; Ni et al., 2020; Wang et al., 2023).

Considering sugar transport, *RabVe_18* contained a SWEET sugar transporter locus (Vitis18g01357). Such transporters have a role in sugar transport during ripening and influence cell expansion, vacuolar filling, and turgor maintenance, thereby indirectly affecting berry firmness (Zhang et al., 2019; Ren et al., 2020).

Additional texture-related genes in this cultivar might include three Wall-Associated Kinases (WAKs; Vitis17g00493, Vitis17g00495, Vitis17g00499), located within *RabVe_17_5* CI and belonging to a class of proteins involved in cell wall–plasma membrane communication and pectin metabolism during fruit development (Seifert and Blaukopf, 2010; Kohorn and Kohorn, 2012).

In Sultanina, as mentioned in Results, QTLs on LG18 include *VviAGL11*, *VviAGL12*, and the *OPR* gene cluster. *VviAGL11* is considered the best candidate for Sultanina, as it plays a key role in ovule identity, seed coat formation, and endosperm development (Smaczniak et al., 2012; Shah et al., 2022; Crespan et al., 2021). Mutations in its promoter cause differential expression between seeded and seedless genotypes, as observed between Sultanina and its seeded variant Sultanina Monococco (Ocarez and Mejía, 2016; Royo et al., 2018). Moreover, the silencing of *SlyAGL11* in tomato led to seedless fruits (Ocarez and Mejía, 2016).

Raboso Veronese and Sultanina share potential regulatory mechanisms for berry texture located on LG11. These include the glycosyltransferases Vitis11g01033 and Vitis11g01296, the cytochrome P450 Vitis11g01052 and the leucine-rich repeat protein Vitis11g01372, already described in Results, and the SBP-domain transcription factor Vitis11g01035. The latter belongs to the SQUAMOSA PROMOTER BINDING PROTEIN (SPL) family, whose members modulate fruit development and ripening through interactions with the miR156 regulatory network (Cui et al., 2018). SPL transcription factors are also recognized as key regulators of ripening and softening in tomato and sweet cherry (Silva et al., 2014; Lai et al., 2020; Sun et al., 2023).

With specific reference to Sultanina, *Sul_11_1* and *Sul_11_2* include the *FAS1* (fasciclin-like arabinogalactan protein) Vitis11g00949. Two Fasciclin-like arabinogalactan genes, named respectively as *VviFLA7* and *VviFLA7a*, were considered among the best candidates affecting berry texture for *MesF11.1*, *MesF11.3*, *PPH11.2*, and *PPH11.3* (Lin et al., 2023), corresponding to QTLs on LG11 detected in this work. However, in the new release of grapevine genome, only the *FAS1* locus Vitis11g00949 was annotated inside the CIs of *Sul_11_1* and *Sul_11_2*. Fasciclin-like arabinogalactan proteins affect cellulose deposition, strengthening plant stem and contributing to the stem elasticity acting on the integrity of the cell-wall matrix (MacMillan et al., 2010).

Overall, our *in silico* analysis identified loci, in addition to *VviAGL11*, within the QTL CIs with annotated functions compatible with roles in berry texture and seed development status. These findings reinforce the association of the corresponding genomic regions with the phenotypic variation observed in this population and highlight potential regulators of the traits under study. While transcriptional and functional validation will be necessary to confirm their specific roles, our study provides valuable targets for future in depth investigation and functional validation.

### 4.4 QTL number inflation for TPA traits and link between seed development status and berry traits

Loooking at the pairwise correlation analysis results (**Figure 3**), we found positive values between three TPA parameters (BH, BG and BCh) and berry dimension (ABW), while the other three (BR, BCo and BS_ratio) showed no correlation.

Therefore, the three dimensionless TPA traits were mapped as they were, while the other three also after normalization, totaling 15 measures. As for BC, ABW and all seed-related traits, excluding the unmapped S, only one QTL was found for each trait. Differently, QTL mapping of texture traits yielded more complex results as multiple QTLs were found to co-segregate with texture traits, mapping also on different chromosomes (**Figure 6**, **Table 5**, **Supplementary File 3**).

In both parental genotypes TPA traits tended to co-segregate with the same QTL or overlap loci sharing the same peak marker, supporting the hypothesis of shared genetic regulation.

QTLs for 5/6 un-normalized TPA traits were mapped in both parents (except BS_ratio, mapped only in Sultanina); for the normalized data, 6/9 were mapped in Sultanina and 4/9 in Raboso Veronese, leading to a different extent and number of QTLs in the two parents around overlapping areas in LG17 of Raboso Veronese and LG18 of Sultanina. Looking at the un-normalized parameters (**Table 5**, **Supplementary File 3**), BH associated to *RabVe_17_1*, and BG and BCh associated to *Sul_11_1* and *Sul_11_2*, respectively, are expected to be influenced by berry size, even if a quantitative estimation of the weights of the two variables is not possible.

In *RabVe_17_2* and in *Sul_18_2*, normalized TPA parameters map in the same QTL of the un-normalized ones. The high EPV values found in *Sul_18_2* (from 29.64 to 36.62) underline the strong association between TPA traits and this QTL.

Moreover, in the Sultanina map, different normalizations of the same parameter map on different QTLs: the normalizations by the diameter for BH, BG and BCh map on *Sul_18_5*, those by the volume map in *Sul_18_2* and *Sul_18_3*, showing shifts around close or overlapping areas. Interestingly, QTLs for normalized BG, BCh and BH in Raboso Veronese, which are closely related to each other (**Figure 4**), even with different CIs, share the same peak marker, while QTLs for BR and BCo, which represent the other side of the same coin, map more or less in the same area of LG17 in Raboso Veronese.

Finally, the effect of normalization on EPV values is not predictable; in other words, normalization does not necessarily produce EPVs higher than those of un-normalized parameters (**Table 5**, **Supplementary file 3**); anyway, the highest EPV values for TPA traits were found in Sultanina map.

Based on grapevine physiology, seeds are metabolic centres for hormonal signals (auxins, citokinins and gibberellins) that influence pericarp development (Conde et al., 2007) and berry ripening (auxins and abscisic acid) (Wang et al., 2019). In the present study we highlighted co-localization of QTLs for seed and berry traits. Even though QTL colocalization cannot distinguish causal genes from linked ones, we hypothesize that the absence of grape seeds or the presence of defective seeds, with limited, partial, or incomplete development, could alter hormonal signals and that this phenomenon may have an indirect influence on pericarp tissues and, therefore, on berry texture. Of course, since the evidence is limited to QTL colocalization, these are only putative functional relationships, linked to the genetic background under study, that require further validation, as with current data it is not possible to establish a causal relationship between seed development status and berry texture, nor to define the underlying mechanisms.

The highest expression of *VviAGL11* is found in seeds at the pre-veraison stage and targets genes involved in hormone signalling and secondary metabolism during seed development (Amato et al., 2022). Even maternal tissues (unfertilized ovules) can perform this function (Amato et al., 2022), but with less efficiency; hence the well-known lower ABW of seedless grapes.

In our F_1_ population the genotypes heterozygous for the nonfunctional *VviAGL11* allele inherited from Sultanina are not really seedless but show different degree of defective seed development. This means that the portion of genome inherited from Raboso Veronese compensates for the defect, in a quantitatively variable manner, depending on the cross recombination. This phenomenon proves that the effect of the seedless allele of *VviAGL11* depends on the genetic background in which it operates. Its action is simply favoured in the context described by Ocarez and Mejía (2016), who generalized the dominant behaviour of this allele, even if their F_1_ population was obtained by crossing two seedless varieties (Sultanina × Ruby seedless).

## 5 Conclusion

Berry texture and seedlessness are major determinants of grapevine fruit quality, influencing both consumer perception and technological performance.

This study represents the first application of Single Primer Enrichment Technology (SPET) for linkage mapping in grapevine, integrating high-density genotyping with an automated, high-throughput phenotyping platform to dissect the genetic architecture of berry texture and seedlessness.

Despite moderate map inflation likely due to residual genotyping inaccuracies inherent to highly heterozygous genomes, strong collinearity between genetic and physical marker positions, together with the correspondence with previously characterized loci involved in berry and seed traits, supported the robustness of QTLs localization.

Overall, this work demonstrates that, even with some limitations, SPET-based high-resolution linkage mapping combined with high-throughput phenotyping was suitable in building genetic maps in the highly heterozygous grapevine genome and provided new information to dig the genetic mechanisms underlying specific berry and seed traits, offering valuable targets for functional validation and marker-assisted breeding.

High throughput phenotyping and the two parental maps allowed us to highlight a different dynamic of berry texture evolution in the different genetic backgrounds, showing there is a link between seed development status and berry softening during ripening.

## Data Availability

The variant data for this study have been deposited in the European Variation Archive (EVA) at EMBL-EBI under accession number PRJEB112499.
