# Supplementary figures and images for: A comprehensive SPET-based linkage mapping for berry texture and defective seed development in grapevine
Source: Front Plant Sci. 2026 May 15;17:1804241. doi: 10.3389/fpls.2026.1804241 (PMC13219353; doi:10.3389/fpls.2026.1804241)

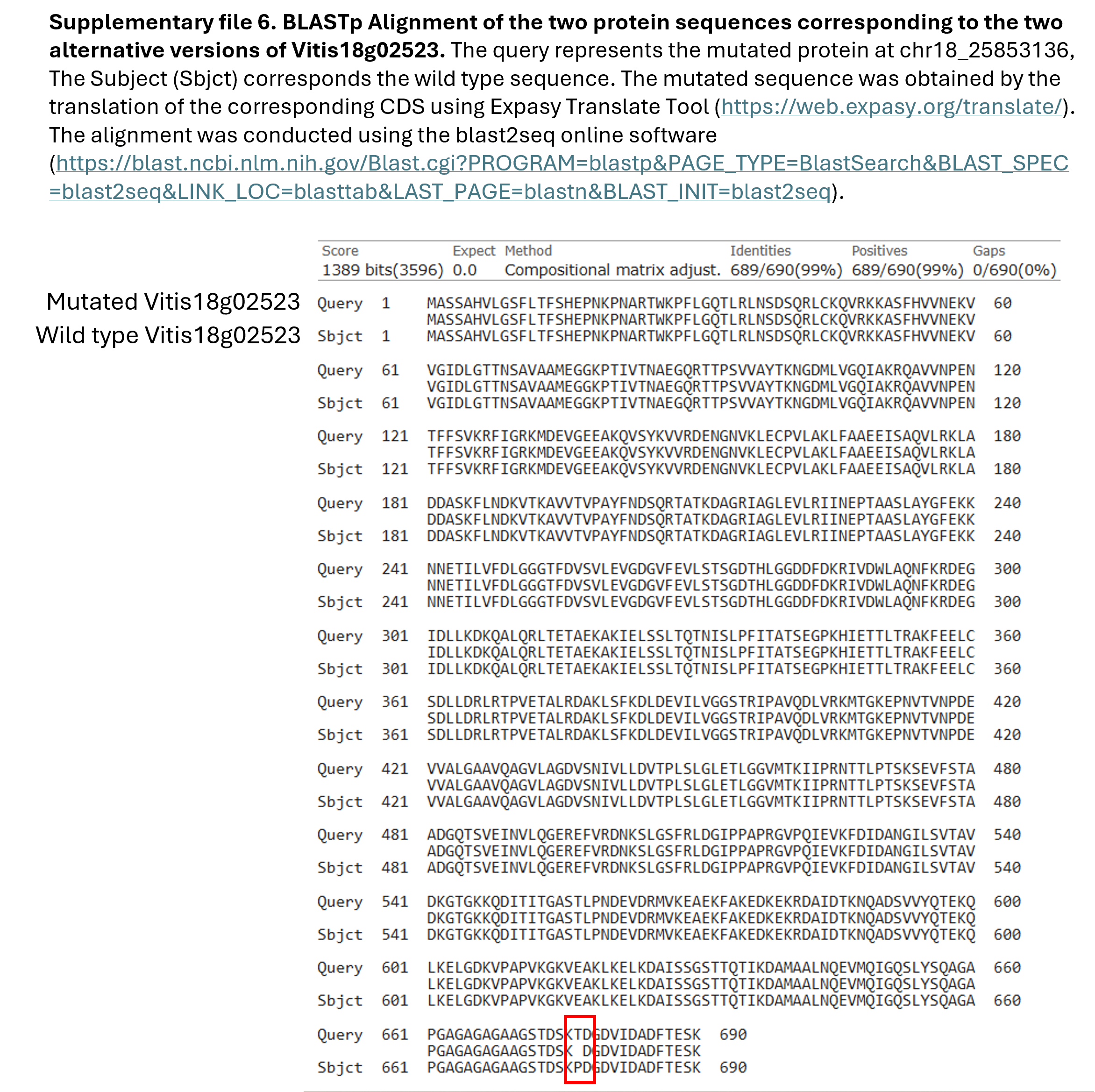

Supplement: Supplementary file 1 [file Image1.tif]

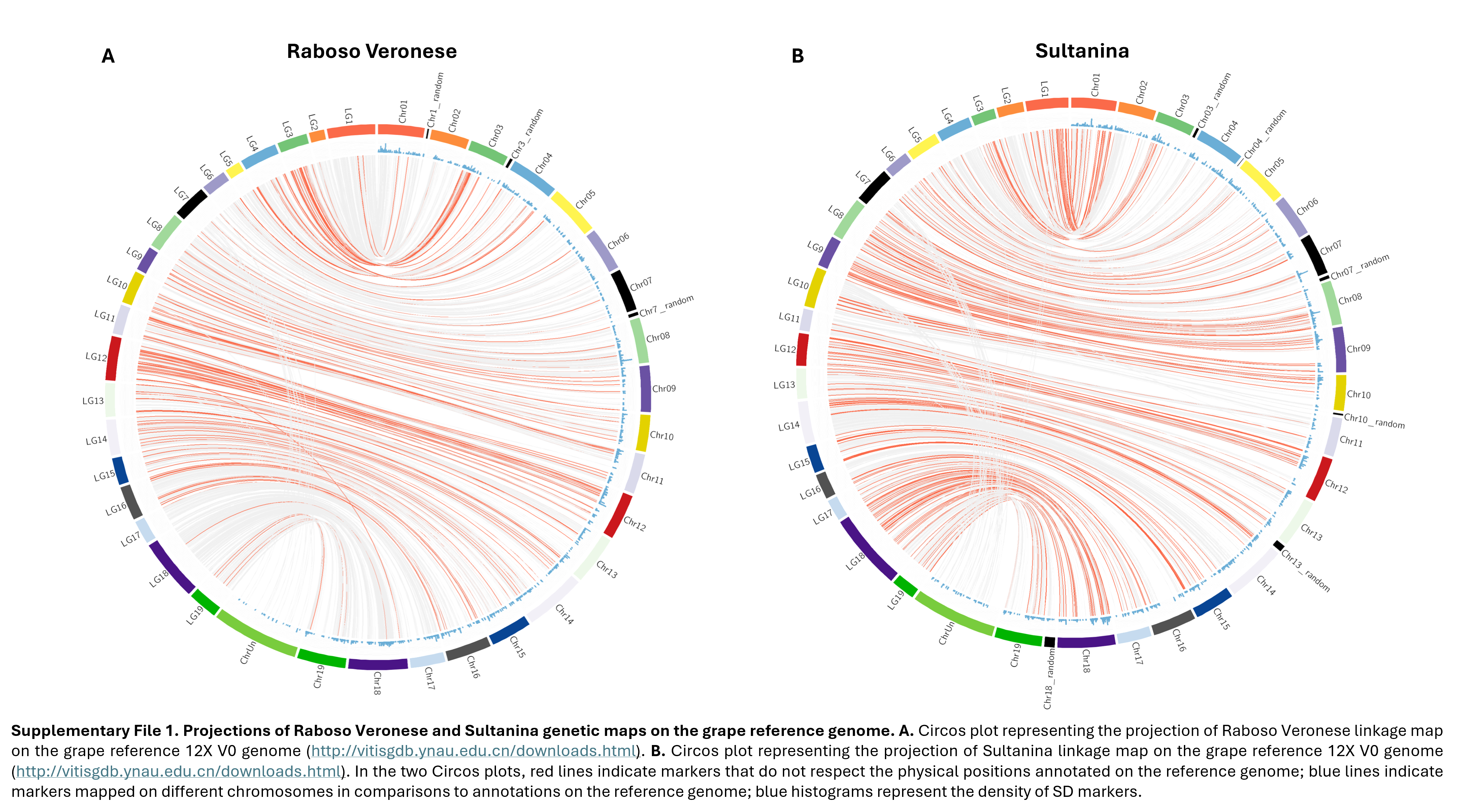

Supplement: Supplementary file 2 [file Image2.png]
