# Supplementary material for: A comprehensive SPET-based linkage mapping for berry texture and defective seed development in grapevine
Source: Front Plant Sci. 2026 May 15;17:1804241. doi: 10.3389/fpls.2026.1804241 (PMC13219353; doi:10.3389/fpls.2026.1804241)
Supplement: Supplementary file 5 [file Table3.docx]

##### **Supplementary file 4.** **Summary of the number of annotated genes identified inside the CIs of each QTL on the Ensembl database.** For each QTL, the number of loci annotated on InterPro databse is also reported.

| **QTL** | **n. of annotated genes** | **n. of loci with annotated function** |
| --- | --- | --- |
| ***RabVe_2*** | **480** | **93** |
| *RabVe_17_1* | 209 | 185 |
| *RabVe_17_2* | 41 | 35 |
| *RabVe_17_3* | 317 | 280 |
| *RabVe_17_4* | 279 | 248 |
| *RabVe_17_5* | 149 | 134 |
| **Tot. in Raboso Veronese LG17** | **357** | **318** |
| ***RabVe_18*** | **105** | **92** |
| *RabVe_11_1* | 454 | 361 |
| *Sul_11_1* | 423 | 342 |
| *Sul_11_2* | 583 | 471 |
| **Tot. in LG11** | **583** | **471** |
| *Sul_18_1* | 169 | 146 |
| *Sul_18_2* | 169 | 146 |
| *Sul_18_3* | 169 | 146 |
| *Sul_18_4* | 124 | 105 |
| *Sul_18_5* | 169 | 146 |
| *Sul_18_6* | 169 | 146 |
| *Sul_18_7* | 37 | 36 |
| *Sul_18_8* | 3 | 3 |
| *Sul_18_9* | 37 | 36 |
| *Sul_18_10* | 169 | 146 |
| *Sul_18_11* | 169 | 146 |
| *Sul_18_12* | 37 | 36 |
| *Sul_18_13* | 169 | 146 |
| **Tot. in Sultanina LG18** | **169** | **146** |
